# Early Cellular Responses of Prostate Carcinoma Cells to Sepantronium Bromide (YM155) Involve Suppression of mTORC1 by AMPK

**DOI:** 10.1038/s41598-019-47573-y

**Published:** 2019-08-08

**Authors:** David Danielpour, Zhaofeng Gao, Patrick M. Zmina, Eswar Shankar, Benjamin C. Shultes, Raul Jobava, Scott M. Welford, Maria Hatzoglou

**Affiliations:** 1 Case Comprehensive Cancer Center Research Laboratories, The Division of General Medical Sciences-Oncology, Case Western Reserve University, Cleveland, OH 44106 USA; 2 Department of Pharmacology, Case Western Reserve University, Cleveland, OH 44106 USA; 3 Department of Urology, University Hospitals of Cleveland, Cleveland, OH 44106 USA; 4 Department of Biochemistry, Case Western Reserve University, Cleveland, OH 44106 USA; 5 Department of Radiation Oncology, Case Western Reserve University, Cleveland, OH 44106 USA; 6 Department of Radiation Oncology, University of Miami, Coral Gables, FL 33136 USA; 7 Department of Genetics and Genomic Sciences, Case Western Reserve University, Cleveland, OH 44106 USA

**Keywords:** Drug development, Stress signalling, Phosphorylation

## Abstract

The imidazolium compound YM155, first discovered as a potent inhibitor of Survivin, effectively kills many carcinomas in preclinical models. However, the upstream signaling mechanism triggered by YM155 remains unclear. Here we studied early signaling responses *in vitro* in prostate and renal cancer cell lines in a dose-dependent manner. We found that YM155 rapidly activates the retinoblastoma protein, correlating with the loss of expression of all three Cyclin Ds. Using Western blot, various selective chemical inhibitors and q-PCR, we show that YM155-mediated decrease in protein levels of Cyclin Ds, Survivin and Mcl-1 is independent of transcription or proteasomal control mechanisms. Moreover, we provide the first evidence that YM155 changes the phosphorylation status of known mTOR-target proteins involved in translational control, namely ribosomal protein S6 (rS6) and 4E-BP1. Our data support that YM155 achieves this by blocking mTORC1 via the phosphorylation of Raptor at S792 through activated AMPKα (T172). Furthermore, we also used a polysome profile, supporting that YM155 markedly suppresses cap-dependent translation of mRNAs which include Survivin, Cyclin D1 and Mcl-1. We provide the first evidence that YM155 functions as a potent activator of AMPKα, a robust suppressor of mTORC1 and an attenuator of global protein synthesis.

## Introduction

Survivin is a unique member of the inhibitor of apoptosis (IAP) family that plays critical roles in cell cycle and cell survival^1^. This IAP is involved in spindle assembly of normal cells^2^, and entry of cells into the S phase^3^. Survivin is robustly overexpressed in numerous carcinomas, positively correlating with tumor progression and resistance to chemotherapy^4,5^. Consequently, significant research effort has been focused on therapeutic targeting of Survivin in advanced cancers^6^.

Sepantronium bromide (YM155), which is four-ring aromatic heterocyclic cationic imidazolium (MW: 443.3 Da, molecular formula: [C_20_H_19_N_4_O3]^+^ Br^−^) was first described as a Survivin suppressant in a high throughput screen of a small chemical compound library reported to suppress the Survivin promoter in the PC-3 prostate cancer cell line^7^. Sub-nanomolar concentrations of YM155 inhibit Survivin expression on a broad spectrum of cancer cell lines^7^. These include non-Hodgkin’s lymphoma, castration-refractory prostate cancer (CRPC), ovarian cancer, sarcoma, non-small cell lung carcinoma (NSCLC), breast cancer, leukemia and melanoma. Later studies show that YM155 is also a potent suppressor of Mcl-1^8,9^, an anti-apoptotic member of the Bcl-2 family^10^.

Continuous infusion of YM155 in athymic mice bearing PC-3 human prostate cancer xenografts caused tumor regression accompanied by increased apoptosis, decreased mitosis, and depletion of intra-tumoral Survivin, but without significant loss in body weight or overt systemic toxicity^11^. Pharmacokinetic analyses show YM155 concentrates in human tumor xenografts^7^. Owing to the highly positive outcome of these preclinical studies, YM155 quickly entered phase I and II clinical trials. In phase I studies, YM155 was well tolerated when administered as a continuous intravenous infusion^12,13^. In Phase II trials, YM155 showed moderate activity as a single agent on CRPC, NSCLC, and stage III and IV melanoma^14–16^.

Some preclinical tumor studies more recently demonstrate markedly improved efficacy (or synergy) of YM155 in killing tumors when combined with other cancer therapeutics^17–21^. What remains a bottleneck in exploring its full clinical potential is an understanding of YM155’s early biological responses and direct molecular target(s) mediating them. The study of the early and late drug responses will allow the rational design of drugs, using YM155 as a lead compound. Here we provide new insight into the roles of mTOR and AMPK on early signals triggered by YM155.

## Results

### YM155 kills various prostate and kidney cancer cells

We examined the relative potency of YM155 (72 h treatment) in killing various prostate genitourinary cancer cell lines (Table 1, Supplementary Fig. S1A,B), which included the PC-3 AR-negative bone metastatic human prostate cancer (PCa), the AR-negative DU-145 brain metastatic human PCa cell line, the LNCaP androgen-dependent PCa lymph node metastatic cell line, the castration-refractory PCa variant of LNCaP (C4-2), bone metastatic castrate-resistant variant of C4-2 (C4-2B), castrate-resistant PCa cell line derived from a clinically localized tumor (CWR22Rv1), normal human prostate epithelial cells (HPEpiC), immortalized prostate epithelial lines (RWPE-1, RWPE-2), and renal cell carcinoma cell lines (RCC4, 786-O) (Table 1). Among the CRPC cell lines tested, the PC-3 line was the most sensitive to YM155, with an IC_50_ of 1.84 nM, while the androgen-dependent prostate cancer cell line, LNCaP, was the least sensitive (IC_50_ = 44 nM). The CRPC (C4-2) and metastatic (C4-2B) variant of the LNCaP line had intermediate levels of sensitivity to YM155 (Table 1). The immortalized and ras-transformed human prostate epithelial cell lines RWPE-1 and RWPE-2 were half-maximally growth inhibited at about 25 nM and 29 nM YM155, respectively. YM155 also had potent growth inhibitory activity on renal clear cell carcinoma line RCC4 (IC_50_ = 1.5 nM) and was less effective on renal clear cell carcinoma line 786-O (IC_50_ = 16 nM). YM155 also inhibited the growth of prostate epithelial cells (HPEpiC) but with an IC_50_ of 99.6 nM (Fig. S1B). Overall, YM155 effectively kills or inhibits the growth of aggressive PCa cells in the low nM range, and the IC_50_ for growth inhibition is generally lower for the more aggressive PCa lines.

**Table 1:** Effect of YM155, assayed in dose-response curves (6 to 7 dilutions, 3-fold each), on changes in growth of prostate and renal cell lines after 3 days was assessed by crystal violet staining of adherent cells.

Cell Lines	Description	IC_50_ (nM)
PC-3	human AR-negative PCa	1.8
PC-3*	PC-3 treated at confluent density	4.5
DU-145	human AR-negative PCa	6.0
LNCaP	human androgen-dependent PCa	44
C4-2	CRPC variant of C4-2	8.7
C4-2B	bone metastatic variant of C4-2	8.0
22RV1	human CRPC from primary tumor	8.1
HPEpiC*	normal human prostate epithelium	110
RWPE-1	HPV18-immortalized HPEpic	25.3
RWPE-2	Ras transformed RWPE-1	29.2
RCC4	human renal cell carcinoma	1.5
786-0	human renal cell carcinoma	16.0

IC_50_ = half maximal growth inhibitory concentration. *Designates YM155 treatment at near confluent density. The standard errors were <8% of each of the respective means (n = 4).

### YM155 suppresses Survivin expression and activates Rb in prostate and kidney cancer cells

**Figure 1:**
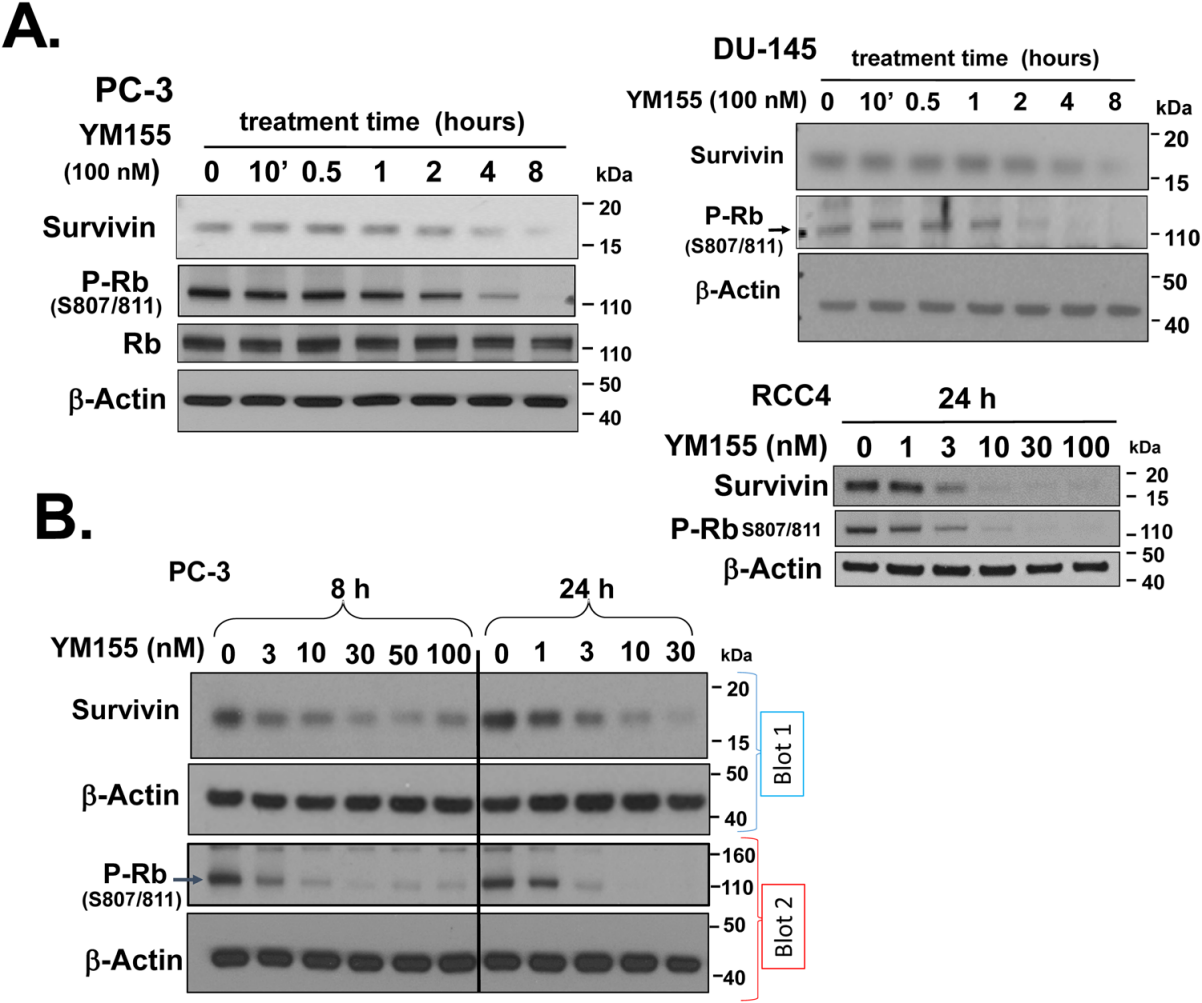
YM155 decreases Survivin expression and activates Rb in prostate and kidney cancer cells. (**A**) PC-3 and DU-145 cells were treated with 100 nM YM155 for various times (10 min – 8 h) as indicated, and expression of Survivin, Rb and p-Rb^ser807/811^ were assessed by Western blot. (**B**) PC-3 cells and RCC4 cells were treated with different concentrations of YM155 for 8 and 24 h, and the expression of Survivin and p-Rb^ser807/811^ were assessed by Western blot. Equal loading and transfer were confirmed by re-probing blots for β-Actin. Unless indicated on the figure, each group of blots represents data obtained from a single gel and experiment. Results shown are representative of 2–3 independent experiments.

The cationic charge of YM155 resulting in a low cLogP (−2.008; partition coefficient) contributes to YM155’s poor cell permeability^22–25^. Cationic transporters are required for cellular uptake of YM155^21,22^. This transport delays the timing of intracellular drug uptake relative to membrane-permeable drugs. Minematsu *et al*.^22^ studied the cellular uptake of [^14^C]YM155 and used the Michaelis-Menten Equation to derive the V_max_ (14.93 pmol/min/mg protein) and K_m_ (0.243 μM) for uptake of YM155 by PC-3 cells. Based on those values we calculated that an intracellular concentration of 3 nM YM155 (IC_50_ of YM155 for killing PC-3 cells in 3 days) is achieved in ~6 h at the K_m_ of YM155. Our data revealed that 100 nM YM155 (41% K_m_) rapidly (1–2 h) activated the Rb protein (as demonstrated by dephosphorylation of Rb at S807/811) in PC-3 cells, which temporally preceded the loss of Survivin protein at 4–8 h (Fig. 1A, left panel). We observed similar effects in YM155-treated DU-145 cells (Fig. 1A, right panel).

We thus studied those alterations in PC-3 and RCC4 cells at various doses of YM155 (1–100 nM) after 8 h and 24 h of YM155 treatment (Fig. 1B). Previous studies in our laboratory showed that Survivin expression is functionally linked to the inactivation retinoblastoma proteins^26,27^. We first focused on this potential link in the mechanism of YM155 action. We found that activation of Rb and loss of Survivin had the same or similar dose-response curves at both times, and such changes occurred within their IC_50_s for cell killing after 72 h (Table 1). These data are consistent with the possibility that YM155 drives the loss of Survivin expression by activating Rb.

Thus, to study early responses of YM155 we used 100 nM YM155 for our subsequent initial studies. This higher dose decreased the lag time of the cationic pump-dependent YM155 uptake.

### YM155 promotes rapid loss of Cyclin Ds

**Figure 2:**
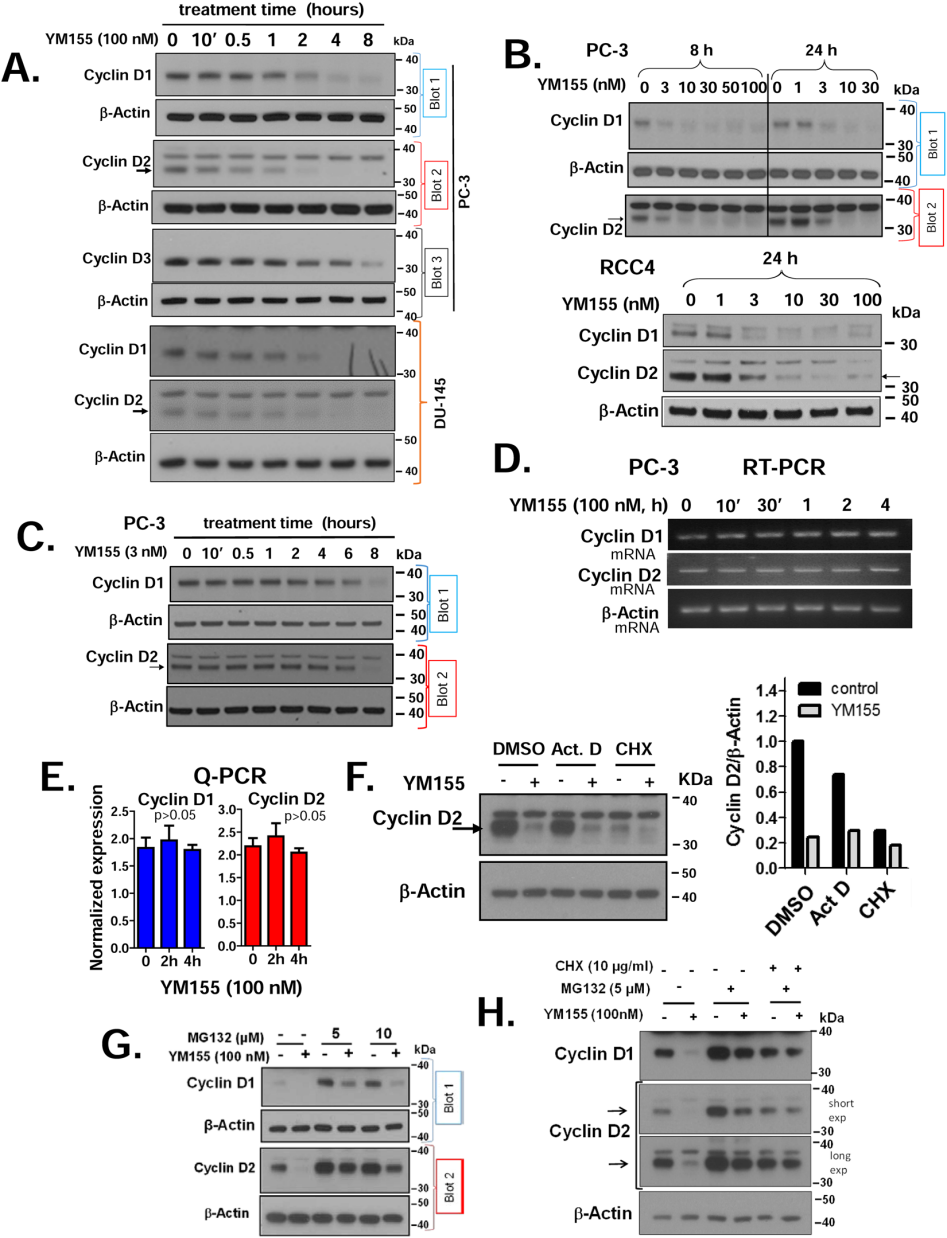
YM155 decreases the expression of cyclin Ds at the protein level: (**A**) We treated PC-3 and DU-145 cells with 100 nM YM155 for the indicated times and used Western blotting to assess the expression of Cyclin Ds 1, 2, and 3. (**B**) PC-3 cells and RCC4 cells were treated with different concentrations of YM155 for 8 and 24 h, and expression of Cyclin Ds 1 and 2 was assessed by Western blotting. (**C**) PC-3 cells were treated with 3 nM YM155 for the indicated times and analyzed as in Fig. 2B. Re-probing blots for β-Actin expression confirmed equal loading and transfer. (**D**,**E**) Expression of Cyclin D1 and D2 mRNA in PC-3 following treatment with 100 nM YM155 were assessed at various treatment times by semi-quantitative RT-PCR (**D**) and real-time quantitative PCR normalized to GAPDH levels (**E**). Results shown are representative of 2–3 independent experiments. (**F**) Effect YM155 (100 nM, 4 h) on suppression Cyclin D2 as assessed in the presence of 2 μg/ml Act D, 2 μg/ml CHX and the DMSO vehicle control (upper panel), as assessed by Western blot and quantified densitometrically using the Image J software (right panel). (**G**) Dose-dependent effect of MG132 as a suppressor of YM155-mediated Cyclin D loss in PC-3 cells, as assessed by Western blot. (**H**) The combined effect on MG132 and CHX pretreatment (30 min) on the influence of YM155 on Cyclin D levels in PC-3 cells after 4 h treatment, as assessed by Western blot. Unless indicated on the figure by blot numbers, each group of blots represents data obtained from a single gel and experiment. Results shown are representative of 2–3 independent experiments.

We next explored the mechanism by which YM155 may dephosphorylate Rb. The Cyclin D-CDK complexes are key enzymes that inactivate Rb through directly phosphorylating Rb and other pocket proteins, thereby releasing the E2F factors that initiate the transcription of several genes required for the entry of cells into the S phase of the cell cycle^28^. A common mechanism of such regulation is through changes in cyclin D levels. Thus, we measured changes in levels of Cyclin Ds in prostate and kidney cancer cells following YM155 treatment. We observed that YM155 (100 nM) caused loss of Cyclin D1 as early as 1 h, and Cyclin D2 as early as 30 min of treatment in the PC-3 and DU-145 cells (Fig. 2A). In the PC-3 and RCC4 cells, Cyclin D1 and D2 expression were decreased by 8 to 24 h with as low as 3 nM YM155, a concentration within its IC_50_ for cell death after 3 days (Fig. 2B). We performed a time course of those changes with 3 nM YM155 on PC-3 cells and found YM155 decreased Cyclin D1 and D2 expression following 2 h of treatment (Fig. 2C).

We subsequently examined whether loss of Cyclin Ds by YM155 was driven by a transcriptional versus a post-transcriptional mechanism. We assessed mRNA levels of Cyclin Ds by both semi-quantitative (Fig. 2D) and real-time quantitative RT-PCR (Fig. 2E) following treatment of PC-3 cells with YM155. YM155 did not alter Cyclin D mRNA levels under conditions that caused robust loss of Cyclin D proteins (examined side-by-side), indicating that YM155 suppresses the expression of Cyclin Ds mainly at the protein level. We tested this further by treating PC-3 cells with sufficient concentrations of either the transcriptional inhibitor actinomycin D (Act D) or the translational inhibitor cycloheximide (CHX) that entirely block transcription versus translation at the elongation step before YM155 treatment. Such abrogation for 4 h of YM155 treatment did not appreciably reduce the levels of Cyclin D2 or affect the ability of YM155 to suppress Cyclin D2 (Fig. 2F), supporting transcriptional responses on Cyclin D2 cannot explain the loss of Cyclin D2 protein. In contrast, CHX effectively suppressed levels of Cyclin D2 and dampened the response to YM155. These data lend further support that YM155 does not suppress the expression of Cyclin D2 through a transcriptional mechanism or message stability.

**Figure 3:**
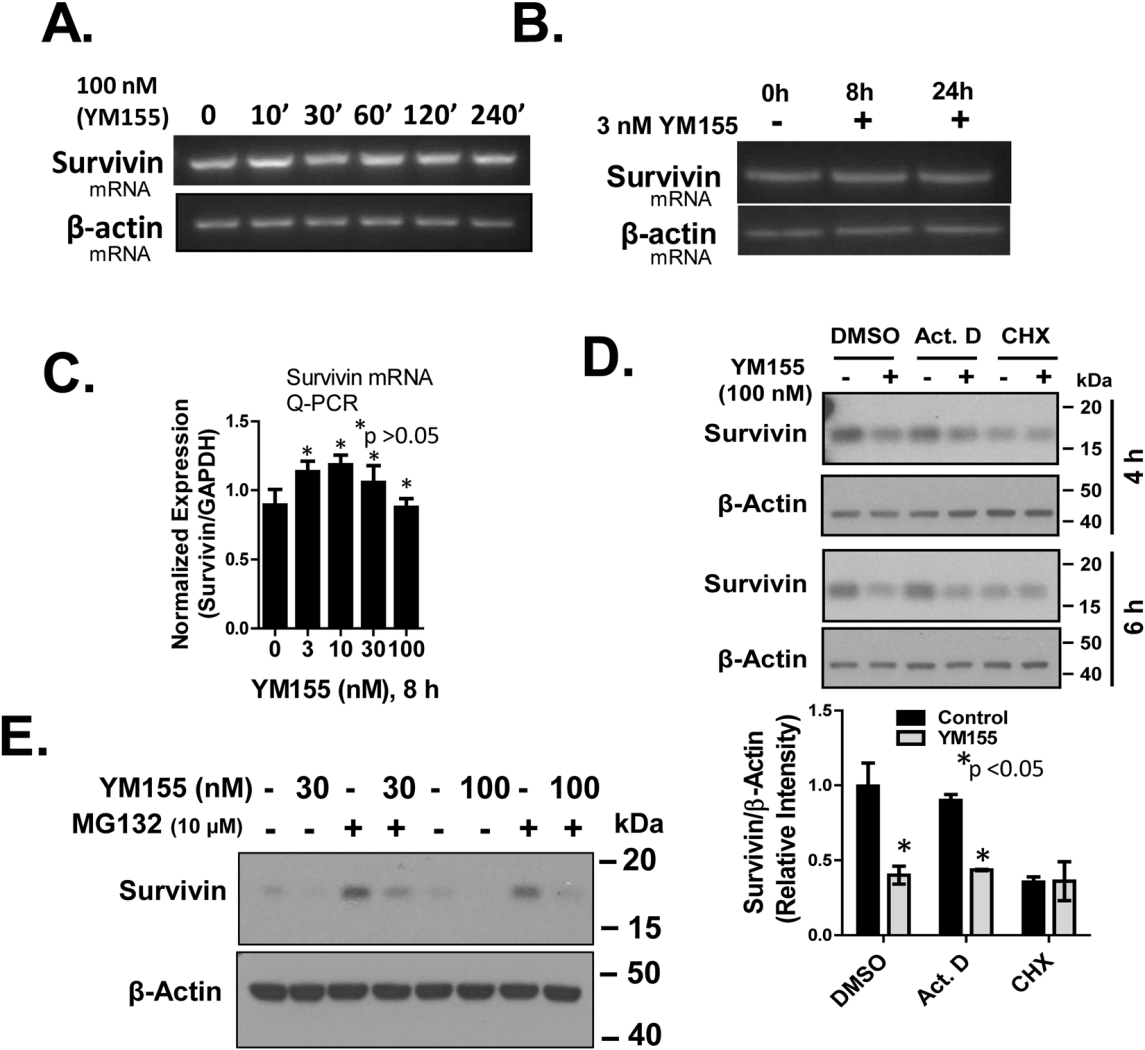
Suppression of endogenous Survivin by YM155 occurs through a non-transcriptional mechanism. (**A**) Effect of 100 nM and 3 nM YM155 the endogenous Survivin mRNA in PC-3 was assessed by semi-quantitative RT-PCR at the indicated times and quantitative real-time PCR at 8 h treatment (**C**). (**D**) Effect of 100 nM YM155 (4 and 6 h) on the expression Survivin in PC-3 pretreated for 30 min with Act D (2 μg/ml) or CHX (2 μg/ml), assayed by Western blot and quantified by Image J analysis. (**E**) Effect of MG132 (10 µM) on the suppression of Survivin by 30 nM and 100 nM YM155 in PC-3, assayed by Western blot. Each group of blots represents data obtained from a single gel and experiment. Results shown are representative of 2–3 independent experiments.

We next tested whether YM155 promoted loss of Cyclin Ds through activating its proteasomal degradation. Pretreatment of PC-3 cells with 5 μM proteasomal inhibitor MG132 did not rescue cells from the suppressive effects of YM155 (Fig. 2G), although it substantially enhanced the basal levels of those Cyclins (evidence that MG132 was effective in blocking proteasomal degradation). Doubling the dose of MG132 in the above experiment failed to rescue the loss of Cyclins by YM155 (Fig. 2G), suggesting proteasomal degradation does not play a major role in the ability of YM155 to drive their downregulation. In another experiment, we re-evaluated the effect of CHX side-by-side with MG132 on the expression of Cyclin Ds (Fig. 3H). Under those conditions, our data support that YM155 suppresses the expression of Cyclin Ds in the absence of MG132, but not when combined with CHX. These data suggest that YM155 may promote loss of Cyclin Ds, particularly Cyclin D1, mainly by inhibiting their translation.

Despite G1 growth arrest signals, cell cycle analysis of PC-3 cells at 24 h with 3 nM and 10 nM YM155 revealed an increased sub-G1 and S phase (Supplementary Fig. S2), consistent with bypassing G1, arresting in S phase and undergoing cell death.

### YM155 suppresses the expression of Survivin through a transcriptional mechanism

Cyclin Ds have been shown to regulate Survivin through a transcriptional mechanism^26,27,29^. However, our semi-quantitative RT-PCR (Fig. 3A,B) and real-time Q-PCR (Fig. 3C), each of which was done side-by-side, showed that YM155 did not alter Survivin mRNA levels. In all of these experiments, we found that doses and treatment times with YM155 that suppressed Survivin protein levels did not suppress Survivin mRNA levels, as confirmed by biological replicates and two different sets of primers (see Material and Methods). Consistent with the above results, we show YM155 inhibited the expression of Survivin in either control or Act D-treated cells. However, YM155 did not further suppress Survivin levels over that inhibited by CHX alone (Fig. 3D). These results suggest that YM155 blocks the expression of Survivin protein independent of the Survivin promoter.

We next tested whether YM155 promoted loss of Survivin expression through activating its proteasomal degradation. PC-3 cells were pre-treated with MG132 for 30 min before a standard 4 h treatment with YM155 and changes in Survivin expression were assessed by Western blot (Fig. 3E). MG132 afforded no significant reversal of Survivin loss by YM155, although MG132 robustly elevated basal levels of Survivin as we expected.

### YM155 suppresses the expression of the anti-apoptotic protein Mcl-1 through a non-transcriptional mechanism

**Figure 4:**
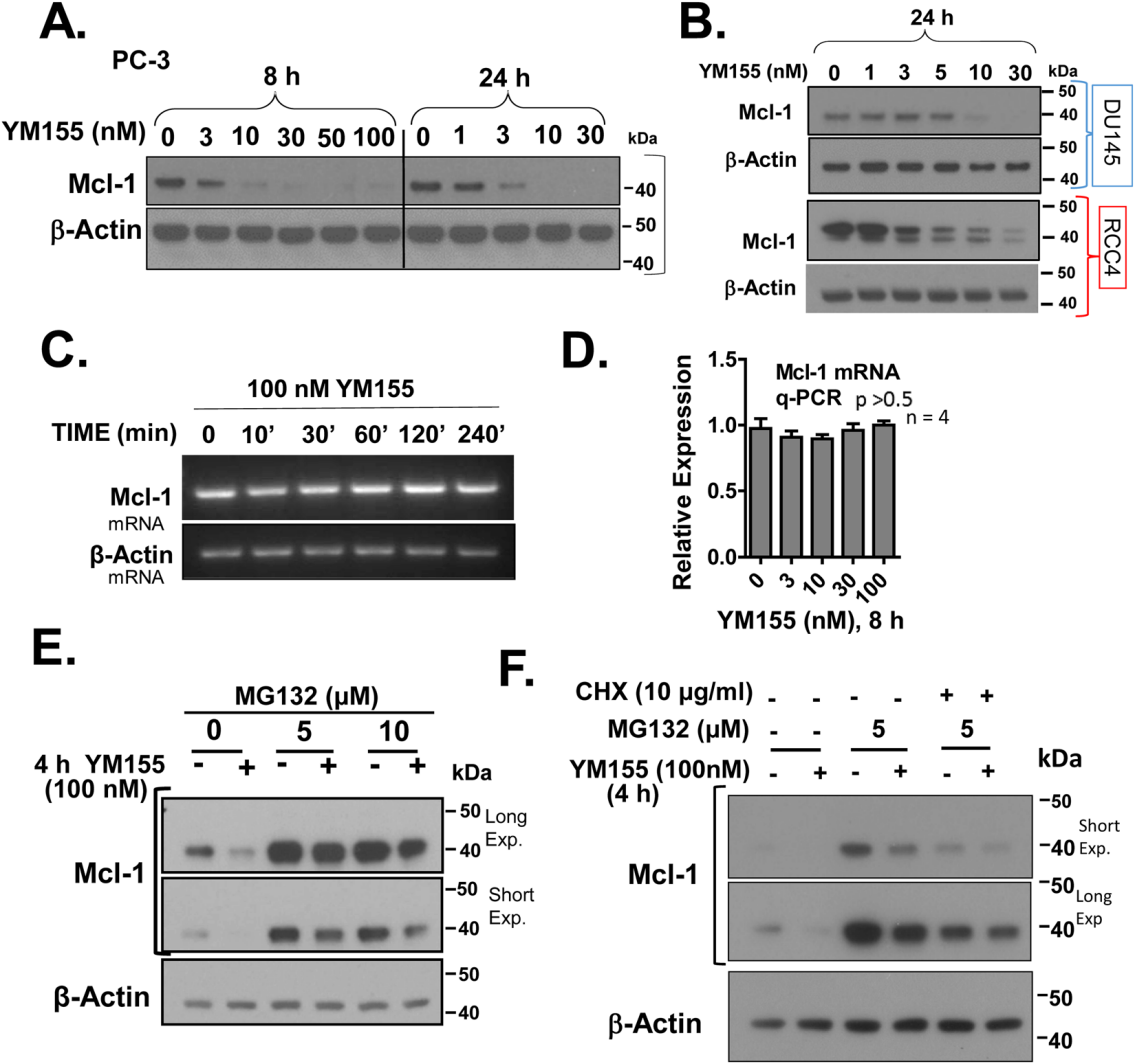
YM155 suppresses Mcl-1 protein levels, but not mRNA levels and such protein may occur partly through a proteasomal-independent mechanism. (**A**,**B**) PC-3, DU-145, and RCC4 cells were treated with the indicated concentrations of YM155 for 8 and 24 h, and Western blotted for Mcl-1 and β-Actin. (**C**,**D**) Effect of YM155 on Mcl-1 mRNA expression in PC-3 was assessed by semi-quantitative RT-PCR (**C**) and quantitative real-time PCR (**D**). (**E**) PC-3 cells were pretreated for 30 min with 5 or 10 µM MG132, followed by treatment with 100 nM YM155 or vehicle for 4 h, after which cells were subjected to Western blotting for expression of Mcl-1 and β-Actin. (**F**) The combined effect of MG132 and CHX (30 min) on the YM155-induced suppression of Mcl-1 in PC-3 cells after 4 h, assessed by Western blot. Unless indicated on the figure, each group of blots represents data obtained from a single gel and experiment. Results shown are representative of 2 to 3 independent experiments.

Mcl-1, an anti-apoptotic member of the Bcl-2 family, has been reported to be robustly suppressed by YM155^8,9,30^. While a transcriptional mechanism of Mcl-1 control by YM155 has been previously proposed^8,9^, the mechanism of such control remains poorly understood. In our time course experiments, we found that treatment of PC-3 cells with 100 nM YM155 can suppress the expression of Mcl-1 by 4 h, with significant suppression at 3 nM and 10 nM YM155 by 8 h and 24 h, respectively (Fig. 4A). YM155 also suppressed the expression of Mcl-1 in DU-145, RCC4 (Fig. 4B), with dose-response patterns comparable to their respective IC_50_s for cell death (Table 1).

We next examined the mechanism by which YM155 suppresses Mcl-1 using PC-3 cells. We assessed levels of Mcl-1 mRNA by semi-quantitative RT-PCR and real-time Q-PCR in PC-3 cells at various times and doses of YM155 treatment that promoted Mcl-1 protein loss, as conducted side-by-side. YM155 did not suppress levels of Mcl-1 mRNA (Fig. 4C,D), as confirmed with biological replicates and two different sets of primers (in Material and Methods), while parallel cultures treated identically responded to YM155 with loss of Mcl-1 protein. These data suggest that YM155 suppresses the expression of Mcl-1 through a non-transcriptional mechanism at those early treatment times.

We next examined whether YM155 promoted loss of Mcl-1 expression through activating its proteasomal degradation. PC-3 cells were pre-treated for 30 min with MG132 before a standard 4 h treatment with YM155, and changes in Mcl-1 expression were assessed by Western blot (Fig. 4E). MG132 (~ 4-fold) stabilized levels of Mcl-1 and appeared to at best minimally block (<30%) its suppression by YM155. To further test the relative roles of the proteasomal and translational controls in the mechanism by which YM155 suppresses Mcl-1, we examined the effect of YM155 on translational control by suppressing protein synthesis at the elongation step with CHX in the absence of proteasomal degradation. Under those conditions, the effect of YM155 on the suppression of Mcl-1 was efficiently voided by CHX in the presence of MG132 (Fig. 4F). These data suggest that YM155 also promotes loss of Mcl-1 by inhibiting its translation. Collectively, these data are consistent with the possibility that YM155 drives loss of Mcl-1 through translational control.

### YM155 rapidly suppresses the phosphorylation of the ribosomal protein S6

**Figure 5:**
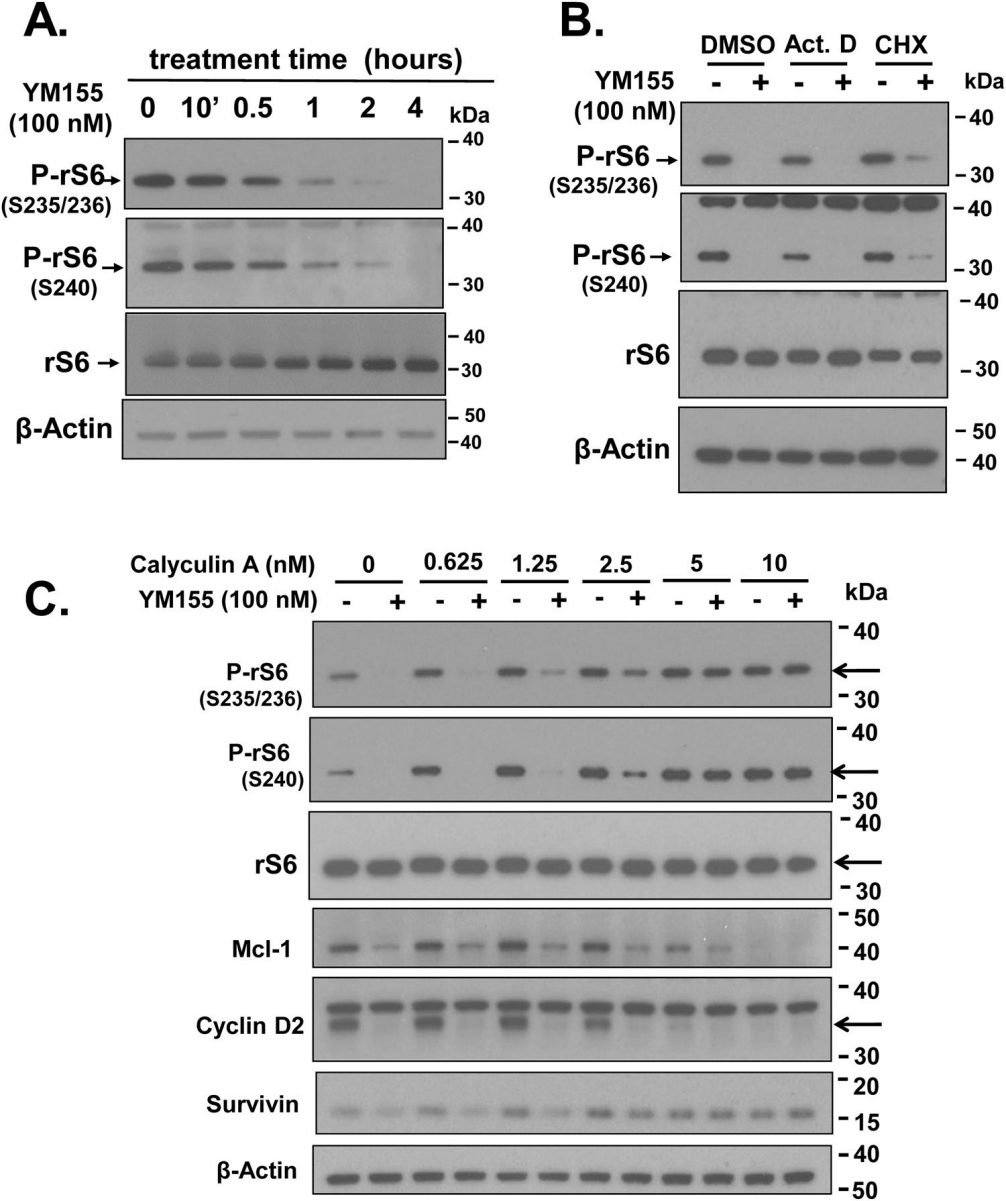
Effect of YM155 on phosphorylation of the ribosomal protein S6. (**A**) Temporal changes in levels of phosphorylated (S235/236) ribosomal protein S6 versus that of total ribosomal protein S6 as assessed by Western blotting. (**B**) Effect of YM155 (4 h) on the suppression of P-rS6(S235/236), P-rS6(S240) in PC-3 cells pre-treated for 30 min with Act D (2 μg/ml), CHX (2 μg/ml) or DMSO vehicle, as assayed by Western blot. (**C**) Effect of YM155 (4 h) on the suppression of Survivin, Mcl-1, Cyclin D2, P-rS6(235/236) and P-S6(S240) in PC-3 cells pre-treated for 30 min with rapamycin (Rap), as assayed by Western blot. Effect of various doses of Calyculin A (30 min pre-treatment) on the suppression of Survivin, Mcl-1, Cyclin D2 and P-S6(S240) by YM155 (100 nM, 4 h) in PC-3 cells, as assayed by Western blot. Unless indicated on the figure, each group of blots represents data obtained from a single gel and experiment. Results shown are representative of 2 to 3 independent experiments.

To examine whether YM155 can suppress the basic, translational machinery common to Survivin, Cyclin Ds, and Mcl-1, we tested the impact of YM155 on the phosphorylation of ribosomal protein S6 (rS6), which plays a vital role in translational control^31^. We found that YM155 rapidly (1–2 h) suppressed the phosphorylation of rS6 at S235/236 in PC-3 cells, with an almost complete loss at 2 to 4 h (Fig. 5A), without suppressing total rS6. Although p70-rS6-kinase (S6K1) phosphorylates rS6 at S235/236, S240 and S244, the ERK and MAPK mitogen-activated kinases can also phosphorylate rS6 at S235/236^32–34^. Thus, we assessed phosphorylation of S240 with a mouse monoclonal antibody as an extra measure of specificity for activation by S6K1. Thus, our data support that YM155 suppresses activation of S6K1. Basal and YM155-suppressed levels of P-rS6 were not affected by Act D or CHX (Fig. 5B), consistent with a protein kinase or phosphatase response. Consistent with this hypothesis, the serine/threonine phosphatase inhibitor calyculin A, which blocks PP1, blocked the dephosphorylation of P-S6 by YM155, but not the ability of YM155 to suppress the expression of Cyclin D2 and Mcl-1 (Fig. 5C). Intriguingly, calyculin A robustly reversed the ability of YM155 to inhibit the expression of Survivin. This phosphatase inhibitor effectively promoted loss of Cyclin D2 and Mcl-1 protein. These results suggest that calyculin A’s ability to reverse YM155’s action on Survivin likely requires a serine/threonine phosphatase(s).

### YM155 suppresses mTORC1 activity

**Figure 6:**
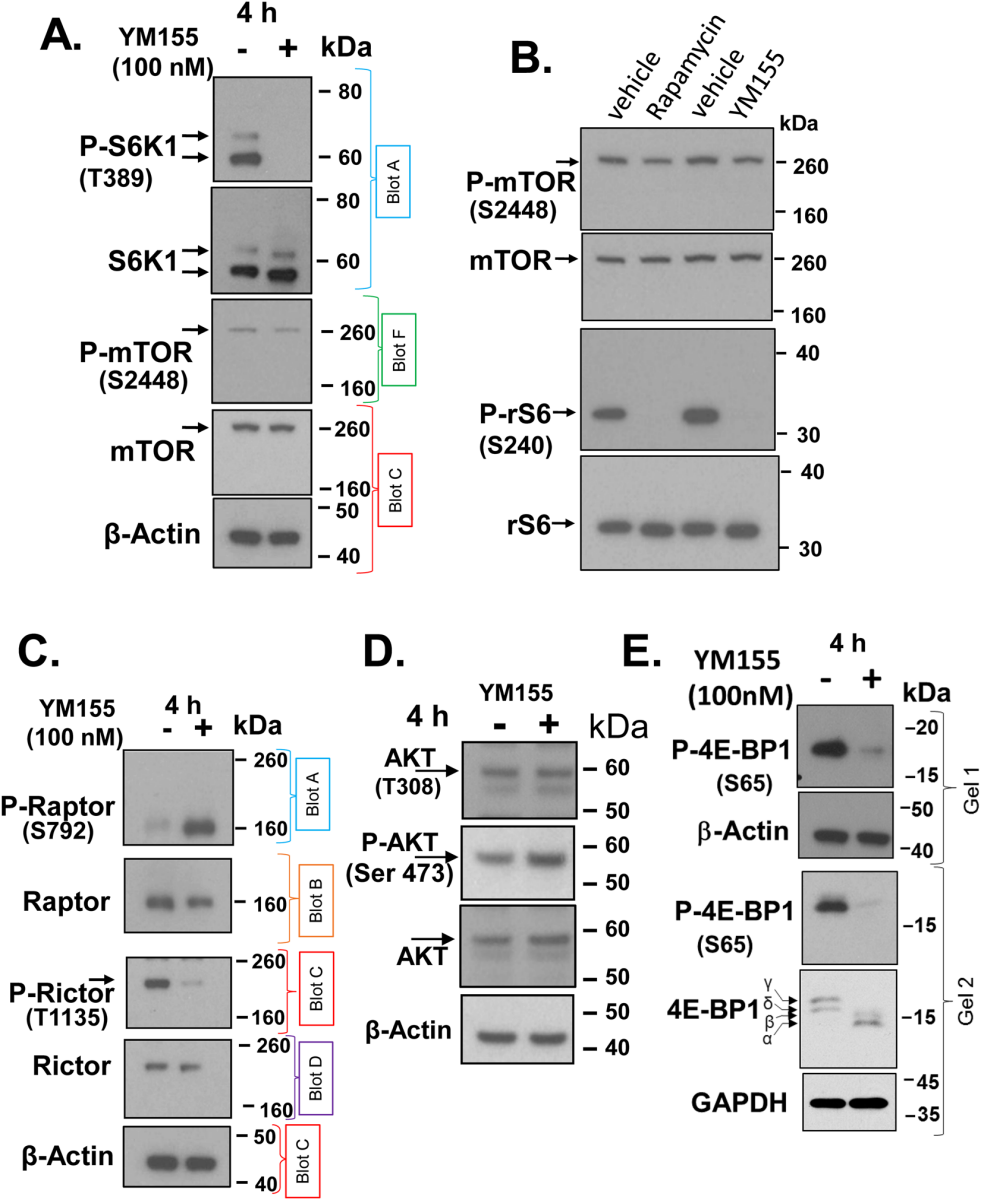
YM155 suppresses mTORC1. (**A**–**E**) PC-3 cells were treated with 100 nM YM155 or vehicle control for 4 h or under the same conditions with 200 nM rapamycin (**B**, lane 2) and changes in total and phosphorylated proteins were assessed by Western blot for S6K1 and mTOR (**A**), mTOR and rS6 (**B**), Raptor and Rictor (**C**), Akt (**D**) and 4E-BP1 (**E**) at the indicated phosphorylation sites. Proteins shown in panels **A**-**C** were electrophoresed through 4–12% Bis-Tris NuPAGE^TM^ using SDS-MOP running buffer (200 V, 4 °C) for better separation of high MW proteins. Analysis of P-4E-BP1(S65) in (**E**) was both through a 4–12% gradient SDS-PAGE (gel 1) and a 15% non-gradient SDS-PAGE (gel 2 Each group of blots represents data obtained from a single gel and experiment, except for P-mTOR and mTOR in (**A**,**C**,**E**) and P-Raptor, Raptor, P-Rictor, Rictor, P-4E-BP1, 4E-BP1 (same sample, protein concentrations, Ponceau S staining, but used different blots to avoid loss of immunoreactivity following blot stripping. β-Actin or GAPDH of the lower blots were used as loading controls). Unless indicated on the figure, each group of blots represents data obtained from a single gel and experiment. Results shown are representative of 2 to 3 independent experiments.

Although our results in Fig. 5 suggested that YM155 inhibited phosphorylation of rS6 through a potential PP1 phosphatase-dependent mechanism^35^, it left open the likelihood that YM155 suppresses the activity of S6K1 (the primary kinase that phosphorylates rS6 at S240^33^). Western blot analysis confirmed that brief treatment of PC-3 cells with YM155 blocked phosphorylated S6K1 at T389 (Fig. 6A), supporting that YM155 suppresses the phosphorylation of S6K1, suggesting it is related to S6K1’s activity^36^.

The major kinase shown to phosphorylate S6K1 at T389 is mTOR in complex 1 (mTORC1)^37^, which is comprised of mTOR and Raptor^38^. We, therefore, examined whether YM155 suppressed mTORC1 kinase by targetting mTOR or Raptor, by Western analysis of phosphorylation sites on mTOR and Raptor (reported to be critical to mTORC1 function)^39^. Treatment of PC-3 cells with YM155 did not alter the levels of total mTOR, although it slightly suppressed P-mTOR (S2448) (Fig. 6A). Of note, studies support that S6K1 rather than Akt is the kinase that phosphorylates mTOR at S2448^40–42^. This suppressive effect was relatively small compared to the robust suppression of S6K1 phosphorylation reported in the literature, suggesting a cell line discrepancy. To test the basis for this discrepancy, we examined the effect of the classical mTOR inhibitor rapamycin side-by-side with that of YM155 in PC-3 cells. Rapamycin, which effectively blocked phosphorylation of rS6 at S240, had a similar effect relative to YM155 on suppressing phosphorylation of mTOR at S2448 (Fig. 6B). This suggests that the limited loss of mTOR phosphorylation at S2448 is perhaps a cell type difference, likely limited by access of the phosphatase that normally removes the phosphorylated residues on mTOR at S2448.

Treatment of PC-3 cells with YM155 induced the phosphorylation of Raptor at S792 (Fig. 6C), while it did not alter the levels of total Raptor. Phosphorylation of Raptor at S792 monitors AMPK activity and AMPK-mediated suppression of mTORC1 functions specifically during energetic stress^39^. In contrast to suppressing mTORC1, our data support that YM155 may possibly activate the mTORC2 complex, as demonstrated by loss of the putative inhibitory phosphorylation site (T1135) on Rictor (Fig. 6C)^43^, an obligate component of the mTORC2 complex^44^. However, the suppression of Rictor activity by its phosphorylation at T1135 generally seems modest and not entirely reproducible^45,46^. Consistent with a potential activation of mTORC2, we found YM155 also slightly increased the phosphorylation of its substrate, Akt (S473) (Fig. 6D), a reported target of mTORC2^44^. YM155 does not seem to significantly alter the levels of total Akt or P-Akt (T308). The putative effect of YM155 on mTORC2 activation is consistent with the potential inactivation of S6K1, as this kinase is known to phosphorylate Rictor^43^.

Consistent with YM155’s inhibition of mTORC1, we found that YM155 also caused loss of the phosphorylation of 4E-BP1 (Fig. 6E), a suppressor of the eukaryotic translation initiation factor eIF4E^47^. Probing the blot for total 4E-BP1, we identified that YM155 affects all four of 4E-BP1’s phospho-variants, most prominently it suppresses phosphorylation of S65. This corresponds to the most massive migrating form, the γ-phosphorylation one^48^ When mTOR is active, phosphorylation of eIF4E by active mTOR kinase causes dissociation of 4E-BP1 from eIF4F, thus enabling the formation of a translationally competent eIF4F complex^49^.

### Impact of YM155 on mRNA translation

**Figure 7:**
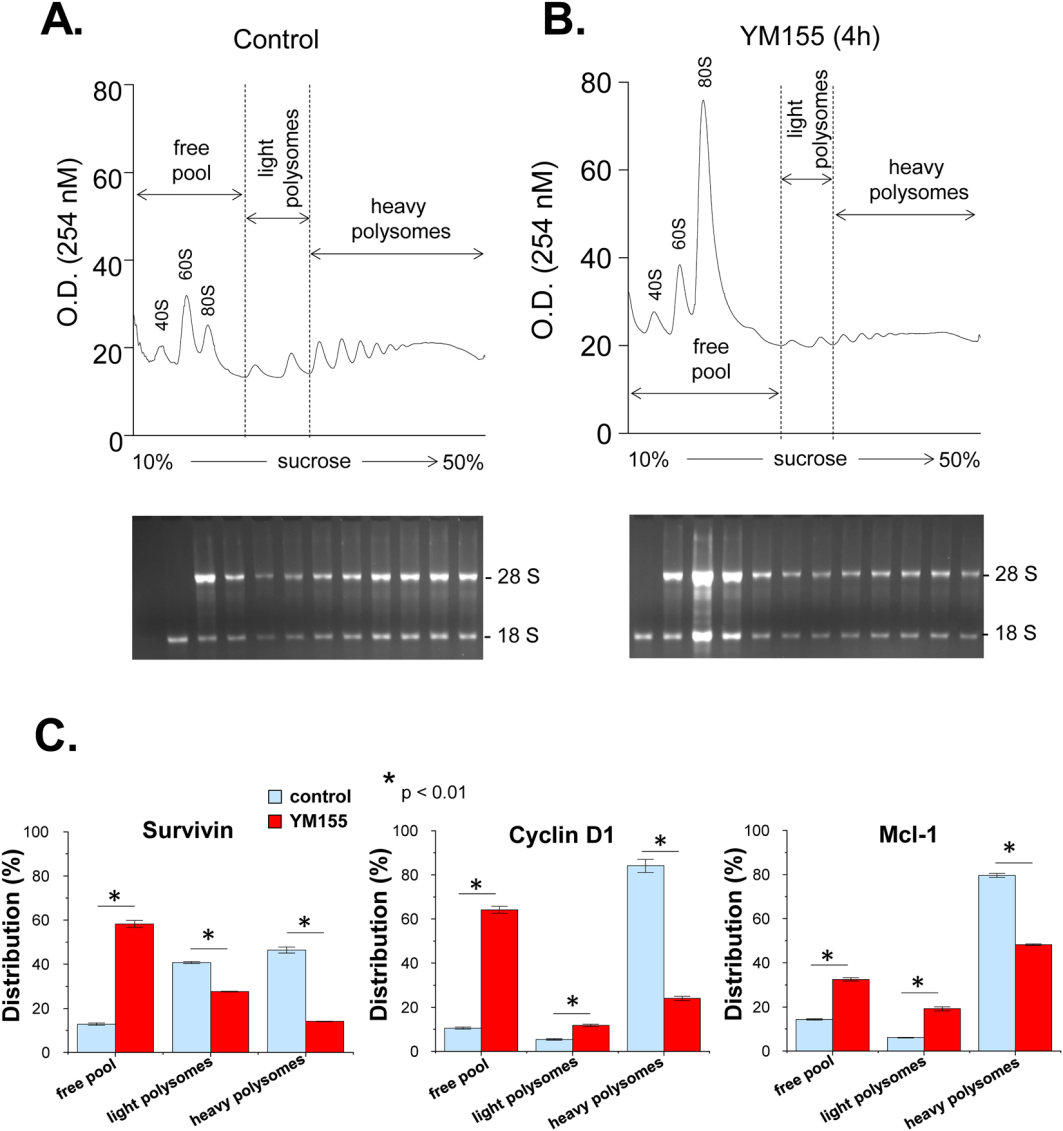
Polysome profile analysis of cells treated with YM155. (**A**,**B**) PC3 cells were treated with DMSO (**A**) or 100 nM YM155 (**B**) for 4 h, and were subjected to the polysome profile analysis. RNA was isolated from each fraction and was visualized via Ethidium bromide-stained agarose gel. (**C**) The association of survivin, cyclin D1, and Mcl-1 mRNAs with the free pool of mRNPs and monosomes, the light polysomes (2- and 3-ribosomes) and the heavy polysomes (>3-ribosomes) were determined by quantitative RT-PCR.

To determine the effect of YM155 on protein synthesis, we treated PC-3 cells with 100 nM of YM155 for 4 h and analyzed global translation by running polysome profiles. The ratio of polysomes to monosomes (5.2 in control and 1.4 in YM155 treatment, respectively) was decreased dramatically by YM155 treatment (Fig. 7A,B, upper panels), which indicated a repressive effect of YM155 on mRNA translation. This is also shown by the observation that YM155 treatment shifted the accumulation of the 28S and 18S ribosomal subunits from the polyribosomes to the free ribosomal pool (Fig. 7A,B, lower panels). The association of Survivin, Cyclin D1 and Mcl-1 mRNAs with the free 80S pool and ribosomes were measured. YM155 treatment caused a severe shift of the Cyclin D1 mRNA from the heavy polyribosomes to the light polyribosomes and the free ribosomal pool (71% decrease in the heavy polysomes, a 2.2-fold increase in the light polysomes, and 6.1-fold increase in the free pool) (Fig. 7C). Translation of the Mcl-1 mRNA was similarly inhibited (39% decrease in the heavy polysomes, a 3.2-fold increase in the light polysomes, and 2.3-fold increase in the free pool (Fig. 7C), although to a lesser extent in the 4 h treatment. The survivin mRNA shifted from both types of polysomes to the free pool (70% decrease in the heavy polysomes, 32% decrease in the light polysomes, and a 4.6-fold increase in the free pool) (Fig. 7C). These data are in agreement with the decrease of the protein levels for these mRNAs (Figs 1, 2, 4). Specifically, Mcl-1 which has a half-life of >30 min^50^, decreased >75% at the levels at 4 h of YM155 treatment (Fig. 4). We conclude that in agreement with the negative regulation of mTORC1 by YM155, this drug has inhibitory effects on protein synthesis and translation of mRNAs that encode for proteins promoting the survival of the prostate carcinoma cells.

### Role of AMPK in the suppression of mTOR by YM155

**Figure 8:**
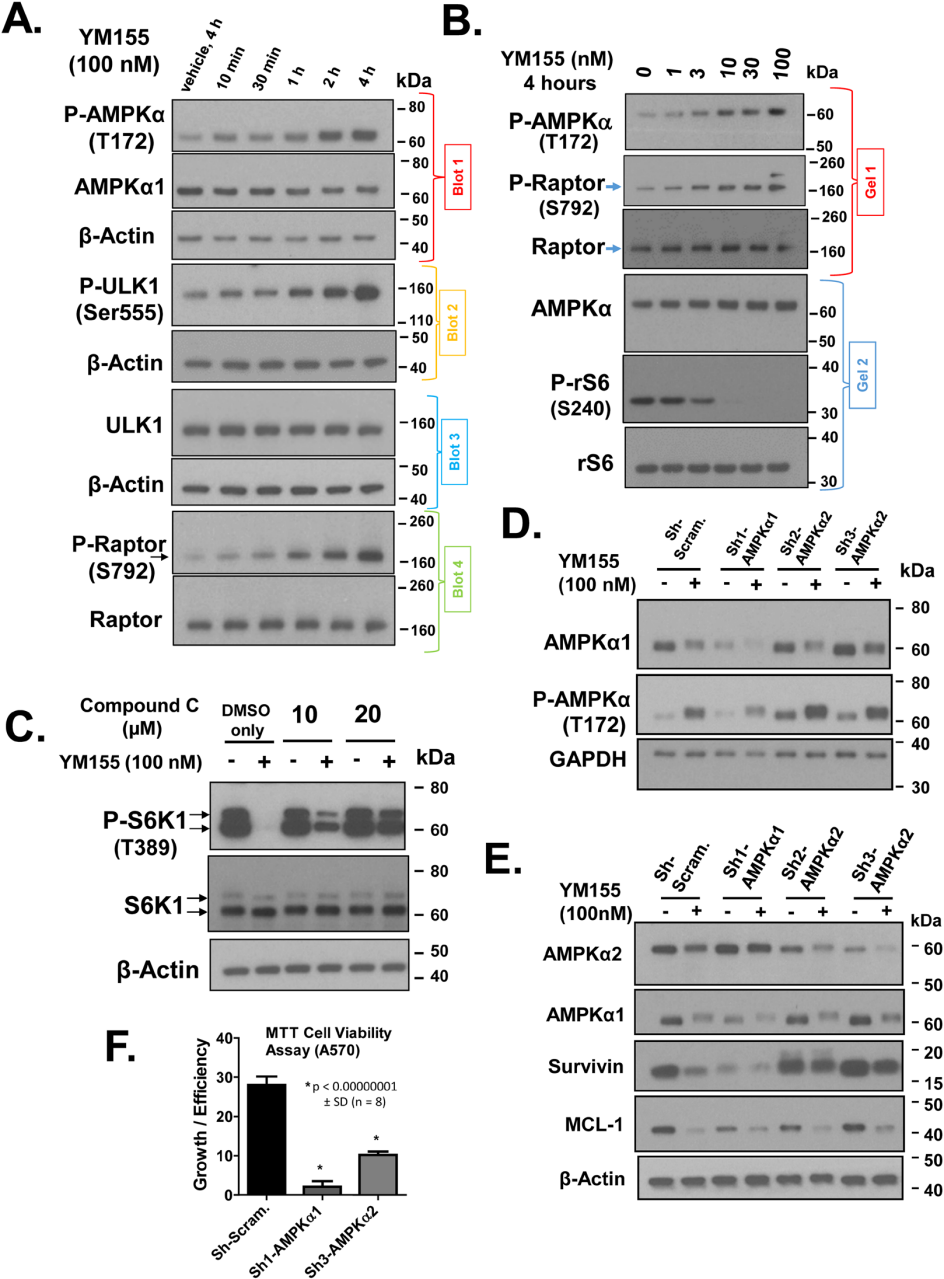
Role of AMPK in cellular responses to YM155. YM155 rapidly activates AMPK in PC-3 cells, and the AMPK inhibitor, Compound C, partially reverses the suppression of P-S6K1 by YM155. (**A**) PC-3 cells were treated with 100 nM YM155 for various early times (from 10 min to 4 h), and changes in the expression of P-AMPKα (T172), AMPKα, P-ULK1(S555), ULK1, P-Raptor (S792), and Raptor were assessed by Western blot. (**B**) PC-3 cells were pre-treated (30 min) with the AMPK kinase inhibitor Compound C or vehicle, followed by treatment with vehicle followed by 4 h treatment with YM155 or vehicle at the indicated drug concentrations, and P-S6K1(T389) and S6K were analyzed by Western blotting. (**C**–**F**) AMPKα1 and AMPKα2 were stably silenced in PC-3 cells by lentiviral-mediated shRNA transduction for 5 to 7 days as described in Materials and Methods. Stably transduced cells were treated for 4 h with 100 nM YM155 or vehicle for 4 h, and the resulting cell lysates were subjected to Western blot for expression of the various protein shown, such as Survivin, Mcl-1, S6K1, PS6K1(T389) P-rS6 (S240) and P-4E-BP1(S65). Knockdown of AMPKα1 or AMPKα2 by lentiviral-mediated shRNA transduction each led to loss of reduced P-S6K1(T3489) changes in P-rS6 (S240) relative to rS6 (**E**) and cell growth/viability by MTT assay (**F**). Relative changes in cell growth/viability of transduced cells by MTT assay as described in Material and Methods. Unless indicated on the figure, each group of blots represents data obtained from a single gel and experiment. Results shown are representative of 2 to 3 independent experiments.

We next examined the mechanism by which YM155 phosphorylates Raptor. Because AMPK is well-established to phosphorylate Raptor at S792^39^, we tested if YM155 can activate AMPK. AMPK is a heterotrimer comprised of a catalytic subunit of AMPKα and two regulatory subunits, AMPKβ and AMPKγ^51^. An ideal marker of activated AMPK is the phosphorylated AMPKα at T172^52^, which we detected using a phospho-specific antibody by Western blot. YM155 (100 nM)-treated cells caused rapid (within 10 to 30 min) phosphorylation of AMPKα at T172 (Fig. 8A). This suggested that YM155 activates AMPK. To confirm that AMPK is active, we examined its ability to phosphorylate its known target, ULK1 at S555^53^, which upon activation is a key inducer of the pathway leading to autophagy. As expected, we found that YM155 promoted the phosphorylation of ULK1 at S555 (Fig. 8A), and the time course of such phosphorylation matched that of the activation of AMPKα (Fig. 8A). Moreover, we showed the other AMPK target protein, Raptor, gets phosphorylated at S792 with similar rapid kinetics as the activation of ULK1 (Fig. 8A). These data, therefore, further support that YM155 rapidly activates AMPK, but such activation may not be direct. However, to the best of our knowledge, the only kinase known to phosphorylate Raptor at S792 is AMPK. Thus, the near temporal coincidence of the phosphorylation of Raptor at S792 within 10 to 30 min of YM155 addition suggests that AMPK directly phosphorylates Raptor, thereby inhibiting mTORC1. To test whether YM155’s ability to active AMPK was limited to a high dose of this drug, we conducted a dose-response study of YM155 on the activation AMPK and suppression of its downstream targets (Fig. 8B). Our results support that 3 nM of YM155 was sufficient to effectively activate AMPK within 4 h treatment, which is within the killing/cytostatic activity of this agent.

We next used Compound C, a selective chemical inhibitor of AMPK^54^, to test our hypothesis that YM155 inhibits mTORC1 through activating AMPK. Focusing on a direct downstream target of mTORC1, we found that pre-treatment of cells with Compound C effectively reversed the dephosphorylation of S6K1 by YM155 (Fig. 8C). These data support our model that YM155 suppresses mTORC1 activity by activating AMPK.

### YM155 differentially affects the activities of AMPKα1 and AMPKα2

There are two isoforms of AMPKα: AMPKα1 and AMPKα2. Each is expressed in normal prostate and prostate cancer tissues, and have unique functions in prostate cancer, either tumor suppressive or oncogenic^55–58^. We, therefore, assessed the role of each of these isoforms in the activity of YM155 by transducing PC-3 cells with lentiviral particles to deliver shRNA for silencing each of those subunits. We achieved >2-fold loss of each isoform, as confirmed by Western blot (Fig. 8D,E). Although silencing AMPKα1 suppressed levels of AMPKα1, it did not effectively block P-AMPKα (T172) activated by treatment with 100 nM YM155 (at 4 h). In contrast, silencing AMPKα2 somewhat enhanced basal and YM155-induced levels of P-AMPKα (Fig. 8D, Supplementary Fig. S3), suggesting that AMPKα1 is the primary AMPKα activated by YM155, and that AMPKα2 functions as a negative regulator of P-AMPKα (T172). This supports our model that YM155 differentially affects each of those two isoforms in PC-3 cells. Intriguingly, although YM155 promoted the phosphorylation of AMPKα, YM155 suppressed the levels of total AMPKα1 and total AMPKα2 (Fig. 8D,E). Moreover, YM155 retarded the migration (through SDS-PAGE) of each isoform, and this change in migration was not observed by another activator of AMPK such as curcumin (Supplementary Fig. S6A). The latter result suggests that YM155 induces an additional modification in AMPKα1 over that by another activator of AMPK. We are not clear what causes loss of AMPKα1 or AMPKα2 levels, as detected by Western blotting. However, such loss is unlikely to be through proteasomal degradation, as MG132 pre-treatment failed to reverse the loss of immunodetectable levels of AMPKα1 (Supplementary Fig. S6B).

We next assessed the effect of silencing AMPKα1 and AMPKα2 on the expression of Survivin and Mcl-1 in response to YM155. Silencing AMPKα1 and AMPKα2 suppressed and enhanced the expression of Survivin, respectively, and silencing each dampened the overall suppression of Survivin by YM155 (Fig. 8E, Supplementary Fig. S5). However, basal levels of Mcl-1 were modestly suppressed and elevated by silencing AMPKα1 and AMPKα2, respectively (Fig. 8E, Supplementary Fig. S5). We also found that knockdown of AMPKα1 or AMPKα2 in PC-3 cells suppressed the relative levels of growth/cell viability compared to control (Fig. 8F). In fact, the AMPKα1-silenced cells were completely growth arrested and could not be expanded under optimal growth. Figure 8F shows growth of those cells by 5 days of culture normalized to their plating density at day 1. These results support that each AMPKα isoform impacts PC-3 cell growth, although AMPKα1 appears to be more critical. Two other constructs we developed to knockout AMPKα1 in PC-3 cells caused the transduced cells to become non-viable, in contrast to the high viability of the respective controls (data not shown). Thus, in PC-3 cells, activation followed by loss of AMPKα1 likely promotes a strong cytotoxic response to YM155.

Importantly and consistent with our data, Sh1-AMPKα1 did not effectively repress the levels of P-AMPKα1(T172) activated by YM155, and both Sh2-AMPKα2 and Sh3-AMPKα2 enhanced P-AMPKα1(T172) (Supplementary Fig. S3). These results support that effective silencing of AMPKα1 and AMPKα2 required to block YM155’s response will repress PCa growth. Thus, a different experimental approach beyond the scope of the current study will likely be required to elucidate mechanistic insight into the role of AMPKs on the action of YM155.

## Discussion

**Figure 9:**
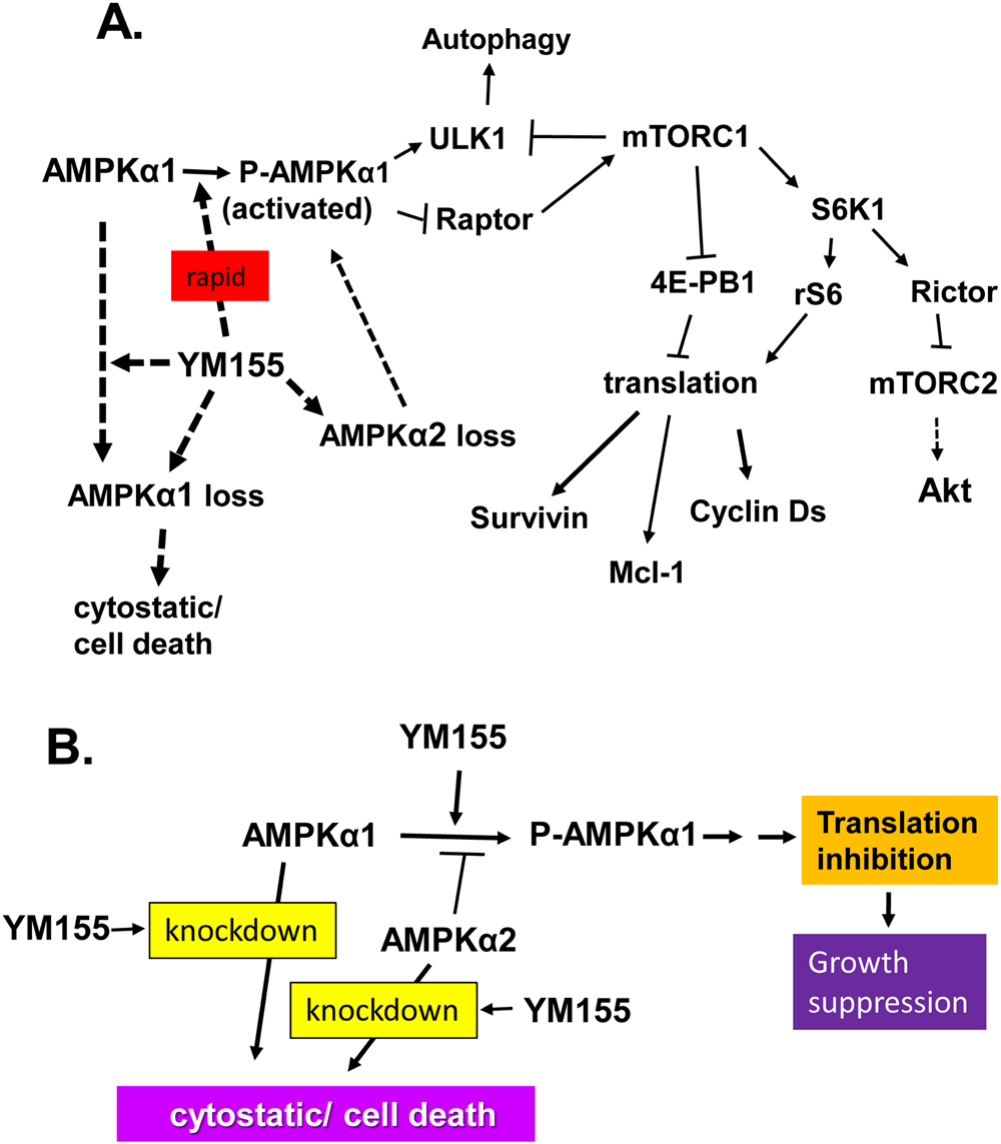
Summary of our model on the molecular action of YM155 in prostate cancer cells. Panel A represents the overall molecular steps in which our model supports that YM155 rapidly promotes the activation of AMPK and subsequent inactivation of mTORC1 and suppression of cap-dependent translation of proteins involved in cell cycle and survival. This model also illustrates that YM155 additionally promotes the loss of AMPKα1 or AMPKα2, and that such loss counter-intuitively promotes growth arrest and cell death. Panel B is a simplified version of panel A.

The mechanism by which YM155 triggers early signals that mediate cell death remains poorly understood. In this study, we provide new mechanistic insight on the early signaling responses of prostate and renal cancer cells to YM155. Figure 9 illustrates a summary of our model. We present the first evidence that YM155 robustly and rapidly inhibits mTORC1 kinase, a critical regulator of cell growth and metabolism. We provide data consistent with a model in which YM155 suppresses mTORC1 through phosphorylation of Raptor by AMPK. YM155 promotes such activation by promoting the phosphorylation of the catalytic subunit of AMPK complexes, AMPKα1 or AMPKα2 at T172. How YM155 promotes such activation is not clear, but it is likely to involve enhanced targeting of LKB1, the key kinase that phosphorylates AMPKα at T172, particularly in PC-3 cells that lack CAMKK2 (88). Although a previous study reported that YM155 induces autophagy in PC-3 cells^59^, consistent with our data on the activation of AMPK and concomitant phosphorylation of ULK1 (Fig. 9A), our study here provides the first underlying mechanism on this problem.

One of the important roles of mTORC1 is to control the translational of a large fraction of cellular proteins, particularly those involved in growth and survival of cells^49^. It is now well recognized that mRNA translation and energy metabolism are dysregulated in cancer and that such dysregulation is a driver of malignancy^60^. mTORC1 controls the translation initiation of those transcripts mainly by phosphorylating S6K1 and 4E-BP1, which activates and inhibits those proteins, respectively. Sabatini’s laboratory conducted global analyses of the rates of mRNA translation in WT and 4E-BP1-knockout MEFs following 2 h of inhibiting mTORC1 with Torin 1 versus vehicle. They identified significant changes in translational control of 4,840 transcripts using transcriptome-scale ribosome profiling^49^. Survivin and Cyclin Ds 1, 2 and 3 were among the transcripts that were translationally suppressed by Torin1 through a 4E-BP1-dependent mechanism (Supplementary Table 1 of^49^). Other studies, using various cell lines, have also demonstrated that mTORC1 controls the levels of Survivin^61^, Cyclin Ds and Mcl-1 through translational regulation^62,63^. These studies are consistent with our model that YM155 suppresses the expression of those proteins partly or entirely through suppressing their translation. Indeed our data in Fig. 7 supports the translation of Survivin, Cyclin D1 and Mcl-1 are significantly suppressed as early as 4 h of YM155 treatment, which is before any noticeable changes in the levels of their transcripts.

Here we established a new paradigm to explain the mechanism by which YM155 causes robust loss of Survivin and Mcl-1. Previous studies claimed that YM155 was a transcriptional suppressor of the Survivin gene. However, our extensive effort (unpublished work) of analyzing the Survivin promoter led us to question that premise and compelled us to postulate an alternative hypothesis that YM155 is not the immediate target of the Survivin promoter. Our hypothesis is clearly supported by the absence of changes in the levels of Survivin mRNA under conditions in which Survivin protein is robustly lost. We showed that YM155 also rapidly suppresses the levels of Mcl-1 through a transcription-independent mechanism since YM155 can suppress Mcl-1 protein without changing its mRNA level.

Although YM155 has been shown to suppress the expression of Survivin mRNA in other systems or conditions, such suppression was relatively small (~50%) compared to a rather robust loss of Survivin protein and appears to require 24 h treatment with high concentrations of YM155^7,11,64^. Though Sp1, ILF3, and p54^nrb^ have been implicated in YM155’s ability to suppress exogenous Survivin promoter constructs^11,65^, their role in suppression of the endogenous Survivin promoter by YM155 is not clear. Plainly, the endogenous promoter functions in the context of chromosomal DNA embedded with chromatin, which is different from exogenous promoters in being chromatin-free.

Even though the potent pro-apoptotic effect of YM155 on cancer cells was ascribed to loss of Survivin^7,66^, YM155 was shown to induce a number of other cytostatic and cytotoxic signals, including inhibition of the phosphorylation of PI3K, ERK1/2, Akt, and STAT3, degradation of EGFR^64^, DNA damage response^67,68^, DNA damage, and oxidative stress^69^. AMPK can be activated by oxidative stress generating peroxides that directly activate ATM, which is the first sensor of the DNA damage response^70^. These data suggest that YM155 may potentially activate AMPK through ATM activated by DNA damage and/or oxidative stress. If so, ATM would need to be activated within 10 min of YM155. Alternatively, due to a potentially unique protein modification of AMPK by YM155 (gel mobility shift), it is possible that the YM155-altered AMPK plays a role in oxidative stress, DNA damage or/and downstream responses.

YM155 has been reported to inactivate Akt through PI3K by blocking upstream receptor tyrosine kinases^64^. However, we do not show any inactivation of Akt after 4 h of YM155 treatment under the conditions where rS6, S6K1, and mTORC1 are suppressed, supporting that YM155 does not block mTORC1 by inactivating Akt or a kinase upstream of Akt. The latter data is consistent with no change in phosphorylation of mTOR at the Akt target site (Fig. 6D).

Our observation that YM155 represses the expression of Mcl-1 is consistent with other carcinomas^8,30^. We are not clear if YM155 drives any proteasomal degradation of Mcl-1, as this drug suppresses Mcl-1 in the presence of excess MG-132 (Fig. 5E,F). Because MG132 robustly stabilizes Mcl-1 in both control and YM155-treated cells, film exposures that detect basal levels of Mcl-1 are often overexposed in lanes represented by lysates of MG132 treated cells even with the inclusion of YM155. In our earlier experiments, we thus initially got the impression that MG132 reverses the suppression of Mcl-1 by YM155. However, film exposures sufficiently low to fall in a linear range show that YM155 still suppresses Mcl-1 even in the presence of excess MG132 and with the same magnitude as without MG132. This suppression is consistent with our data supporting that YM155 suppresses Mcl-1 translational control.

Our data in Table 1 suggests that YM155 is more effective on aggressive prostate cancers compared to less aggressive ones. A good comparison of such response can be achieved with the androgen-dependent LNCaP cell line versus its castrate resistant variant C4-2 along with its castrate-resistant bone metastatic variant C4-2B. Consistent with their IC_50_s for cell death, YM155 more effectively suppressed the expression of Cyclin Ds 1 & 2, Survivin, Mcl-1, and P-S6 levels in C4-2 and C4-2B cells compared with LNCaP cells (unpublished study). Consistent with these results, a recent report demonstrated that androgens enhance the ability of YM155 to kill AR-positive PCa cell lines presumably by inducing the expression of a cationic transporter^21^. Because AR activation promotes the expression of CAMKK2^71^, an AMPKα1 activator, it is possible that the synergistic effect of androgen on the killing of prostate cancer cells by YM155 also or alternatively occurs through enhanced activation of AMPKα by elevated levels of CAMKK2 induced by androgen. Further work is required to explore the role of CAMKK2 in the mechanism by which androgens enhance the ability of YM155 to kill cells and to explore the clinical potential of YM155 and its analogs.

Owing to the fact that PC-3 cells silenced for AMPKα1 are unable to grow (Fig. 8F) and our AMPKα1 knockout clones were non-viable, the function of AMPKα1 in PC-3 cells is consistent with prostate carcinomas^55,56,58^. Although in normal prostate, AMPKα proteins function as tumor suppressors, they have oncogenic functions in prostate carcinoma^55–58^. Data mining from cBioportal and Oncomine support overexpression of AMPKα1 and AMPKα2 in a group of neuroendocrine prostate carcinomas (Supplementary Figs S7, S8). Thus it is likely that AMPK kinase and AMPK protein have different functional roles in prostate cancer cells, such that blocking their kinase is not equivalent to silencing their expression (Fig. 9). In particular, our data support that silencing AMPKα1 or AMPKα2 alone did not noticeably reduce the levels of P-AMPKα (T172), although the loss of AMPKα2 enhanced basal and YM155-induced AMPKα (T172) (Figs 8D, 9, Supplementary Fig. S3). This suggests a potential mechanism by which loss of AMPKα2 may be cytostatic. Importantly, YM155 robustly suppresses the expression of both AMPKα1 and AMPKα2 (Supplementary Fig. S4).

Our model helps predict relevant drug combination of YM155 in prostate and renal carcinomas. As discussed earlier, androgen receptor agonist, would be expected to enhance AMPK activity through overexpression of CAMKK2^55^, and provide the basis for the enhanced killing of AR-positive prostate cancer cells by YM155. Renal cell carcinomas typically lack the tumor suppressor VHL^72^. Functional loss of VHL induces the expression of HIF-1α and subsequent downstream hypoxia response pathway, leading to over vascularization and poor perfusion. We thus recommend moderate inhibition of VEGF to promote vascular normalization and help delivery of YM155.

While our study has addressed many problems, many more remain. What is the direct target of YM155 leading to the activation of AMPK? Does the treatment of cells with YM155 promote them to undergo a unique posttranslational modification of AMPK? How does YM155 cause loss of AMPK expression? Does loss of AMPKα1 and AMPKα2 play a role in the cytostatic/cytotoxic responses of PCa cells to YM155? How can these new attributes be clinically exploited in the design of new potential clinical trials for patients with cancer? As blocking AMPK and mTOR can treat various non-cancer related conditions^73^, can these properties of YM155 raise the potential off-label therapeutic use of this drug and its derivatives?

## Materials and Methods

### Materials

Sources were: YM155 (Selleck Chemicals); rabbit anti-Survivin IgG (#AF886) (R&D Systems); rabbit anti-P-Rb (S807/811, #9308), rabbit anti-P-Cyclin D1 (T286, #3300), mouse anti-Cyclin D1 (#2926), rabbit anti-Mcl-1(#5453), mouse anti-Cyclin D3 (#2936), rabbit anti-P-Akt (T308, #6955), rabbit anti-Akt (S473, #9271), rabbit anti-rS6 (#2217), rabbit anti-P-rS6 (S235/236, #2211), rabbit anti-P-p70S6K1 (T389, #9205), rabbit anti-mTOR (#2972), rabbit anti-P-mTOR (S2448, #2971), rabbit anti-Raptor (#4978, #4972) rabbit anti-P-Raptor (S792, #2083) rabbit anti-Rictor (#2140), rabbit anti-P-Rictor (T1135, #3806), rabbit anti-AMPKα (#5831), rabbit anti-P-AMPKα (T172, #2535) rabbit anti-AMPKα1 (#2795), rabbit anti-AMPKα2 (#2757), rabbit anti-4E-BP1 (#9644), rabbit anti-P-4E-BP1 (S65) (Cell Signaling Technologies); rabbit anti-Rb (sc-50), rabbit anti-Cyclin D1 (sc-753), rabbit anti-Cyclin D2 (sc-593), mouse anti-GAPDH (sc-51907), mouse anti-α-tubulin (sc-5386), mouse anti-P-rS6 (S240, sc-293143), mouse anti-rS6 (sc-74459), mouse anti-p70S6Kα (sc-39367) (Santa Cruz Biotechnologies, Inc.); mouse anti-β-actin (#A-5441), cycloheximide (CHX), actinomycin D (Act D), MG132, curcumin, (Sigma-Aldrich, Inc.); calyculin A, rapamycin and Compound C (LC labs, Inc.) DMEM/F12 (Media Tech); characterized fetal bovine serum (FBS) (HyClone).

### Cell culture

The human PC-3, DU145, LNCaP, CWR22Rv1, RWPE-1, RWPE-2, and 786-0 cell lines were obtained from American Type Culture Collection (ATCC), HPEpiC cells were from Cell Science, Inc. and RCC4 cells were obtained from Sigma-Alrich. PC-3, DU-145, LNCaP, C4-2, C4-2B, and RCC4 cells were maintained in DMEM/Ham’s F-12 medium (1:1, v/v) with 5% FBS, while RWPE-1 and 2 cells were maintained in keratinocyte growth medium supplemented with bovine pituitary extract and 5 ng/ml epidermal growth factor. The LNCaP, C4-2 and C4-2B cells were maintained in plates coated with poly-D-lysine as before^50^. All cell lines were maintained in 5% CO_2_ at 37 °C.

### Cell growth/viability assay

Cells were plated overnight in 12 well plates at a density of 10,000 cells/1 mL/well, treated with YM155 (0.1–1000 nM), and three days later stained with crystal violet, as previously described^27^. The IC_50_ was calculated using GraphPad Prism. MTT assays were also conducted to confirm viability in a 96-well format, using a kit from Sigma-Aldrich as described by their protocol.

### Western blotting

Cells (10^5^ cells/ml DMEM/F12 + 5% FBS) were plated in either 6-well dishes (2 ml/well) or 10-cm dishes (10 ml/dish) for the indicated treatments, and cells were washed, lysed and clarified lysates were processed for Western blot analysis, as detailed before^27^. Unless specified, all proteins for Western analysis were electrophoresed through 4–12% Bis-Tris NuPAGE^TM^ protein gels using SDS-MES running buffer (200 V, 4 °C). Equal loading of samples was based on total protein as determined by a BCA protein assay (96-well format), using an 8-point BSA standard curve, each point in duplicate and samples were the average of 3 replicates determinations. An equal transfer was confirmed by staining membranes with Ponceau S. Chemiluminescent image was captured by autoradiograph on film (Fuji Medical 100 NIF)^27^.

### Reverse transcriptase polymerase chain reaction (RT-PCR)

PureLink^TM^ RNA mini kit (Invitrogen) was used for isolating RNA from cells that were treated with 100 nM YM155 for the indicated time periods. One µg of RNA was reverse-transcribed using M-MLV Reverse Transcriptase (Promega). PCR amplification was performed using Taq-Polymerase Master Mix (Promega) and SYBR Green Real-Time PCR Master Mix (Invitrogen) for semi-quantitative and quantitative real-time PCR, respectively. The PCR primers were: Cyclin D1 Forward: 5′-CAC GGA CTA CAG GGG AGT TT-3′, reverse: 5′-TCT GTT CCT CGC AGA CCT CC-3′; Cyclin D2 forward: 5′-TGC TCT GTG TGC CAC CGA CTT T-3′, reverse: 5′–CGT CTG TAG GGG TGC TGG CT-3′; Survivin (set 1) forward: 5′-TGA CGA CCC CAT AGA GGA ACA-3′, reverse: 5′-CAG TAG AGG AGC CAG GGA CT-3′; Survivin (set 2) forward: 5′-TTC TCA AGG ACC ACC GCA TC-3′; reverse: 5′-TCC TTT GCA ATT TTG TTC TTG GC-3′; Mcl-1 (set #1) forward: 5′- CTT TTG GTG CCT TTG TGG CT-3′, reverse: 5′-TAG TGC TTC TCT TAA CAC TAC TAC A-3′; Mcl-1 (set #2) forward: 5′-GCG ACT TTT GGC CAC CG-3′, reverse 5′-GCT AGG TTG CTA GGG TGC AA-5′; GAPDH forward: 5′-CTC TGC TCC TCC TGT TCG AC-3′, reverse: 5′- AAA TGA GCC CCA GCC TTC TC-3′. AR forward: 5-TAG GGC TGG GAA GGG TCT AC-3′, reverse: 5′-GACACCGACACTGCCTTACA-3′. The products were run on a TAE-1% agarose gel and identified using a Biorad Molecular Imager Gel Doc XR + System or quantified using a Bio-Rad CFX Connect Real-Time PCR Detection System.

### Lentiviral-mediated silencing of AMPKα 1 and 2

Mission pLKO.1 lentiviral shRNA constructs (TRCN0000000861, TRCN0000002170, TRCN0000001271 were obtained from Sigma to silence human AMPKα1 and α2 genes. Viral supernatants were produced in HEK293T cells transfected with pLKO.1 sh-RNA, pMDG, and pCMV-dR8.78. Viral supernatants were tittered for viral levels by an ELISA for p24 using a kit from Sino Biological. PC-3 cells were transduced overnight with viral supernatant (MOI = 0.5) in the presence of 8 μg/ml polybrene, and 24 h after replacing with fresh growth medium, cells selected with 1.5 μg/ml puromycin for 4 days for 100% death of the non-transduced cells.

### Polysome profile analysis

PC-3 cells (70% confluent in two 150-mm culture dishes) were treated with 100 nM YM155 or DMSO for 4 hours. Cells were washed twice with cold PBS, scraped in the lysis buffer (10 mM HEPES-KOH (pH 7.4), 5 mM MgCl_2_, 100 mM KCl, 1% Triton X-100, 5 mM DTT, 100ug/ml Cycloheximide, 200 unit/ml RNase inhibitor (RNaseOUT, Invitrogen), EDTA-free protease inhibitor (Roche Applied Science), gently passed 4 times through a 26-gauge needle and centrifuged at 1,300 x g for 10 min. Approximately 17.5 A_260_ units of lysates were layered over 10–50% cold sucrose gradients in the gradient buffer (lysis buffer without Triton X-100). Gradients were centrifuged at 31,000 rpm in a Beckman SW 32 Ti rotor for 3.5 h at 4 °C. After centrifugation, 12 fractions (1.2 ml/fraction) samples were collected. RNA from each fraction was isolated using TRIzol LS reagent (Invitrogen), and an equal volume of RNA from each fraction was used for cDNA synthesis by qScript^™^ XLT cDNA SuperMix (QuantaBio). The relative quantities of specific mRNAs were measured by reverse transcriptase (RT) - quantitative polymerase chain reaction (qPCR) with the StepOnePlus Real-Time PCR System (Applied Biosystems) using the standard curve method. The qPCR primers are as follows:

MCL-1 forward: 5′- CAAAGAGGCTGGGATGGGTT -3′;

MCL-1 reverse: 5′- AGGTTGCTAGGGTGCAACTC -3′;

Survivin forward: 5′- AGGACCACCGCATCTCTACA -3′;

Survivin reverse: 5′- TGTTCCTCTATGGGGTCGTCA -3′;

Cyclin D1 forward: 5′- AATGACCCCGCACGATTTC -3′;

Cyclin D1 reverse: 5′- TCAGGTTCAGGCCTTGCAC -3′.

## Supplementary information

Supplementary Figures

## Data Availability

Raw data (including full blot images) and experimental details used to generate the final figures in this manuscript will be available upon reasonable request.
