# Author Correction: Early Cellular Responses of Prostate Carcinoma Cells to Sepantronium Bromide (YM155) Involve Suppression of mTORC1 by AMPK

**DOI:** 10.1038/s41598-019-51007-0

**Published:** 2019-10-10

**Authors:** David Danielpour, Zhaofeng Gao, Patrick M. Zmina, Eswar Shankar, Benjamin C. Shultes, Raul Jobava, Scott M. Welford, Maria Hatzoglou

**Affiliations:** 1 Case Comprehensive Cancer Center Research Laboratories, The Division of General Medical Sciences-Oncology, Case Western Reserve University, Cleveland, OH 44106 USA; 2 Department of Pharmacology, Case Western Reserve University, Cleveland, OH 44106 USA; 3 Department of Urology, University Hospitals of Cleveland, Cleveland, OH 44106 USA; 4 Department of Biochemistry, Case Western Reserve University, Cleveland, OH 44106 USA; 5 Department of Radiation Oncology, Case Western Reserve University, Cleveland, OH 44106 USA; 6 Department of Radiation Oncology, University of Miami, Coral Gables, FL 33136 USA; 7 Department of Genetics and Genomic Sciences, Case Western Reserve University, Cleveland, OH 44106 USA

Correction to: *Scientific Reports* 10.1038/s41598-019-47573-y, published online 08 August 2019

In Figure 9A, the connection between S6K1 and Rictor was switched with that between Rictor and mTORC2. The correct Figure 9 appears below as Figure 1.

**Figure 1:**
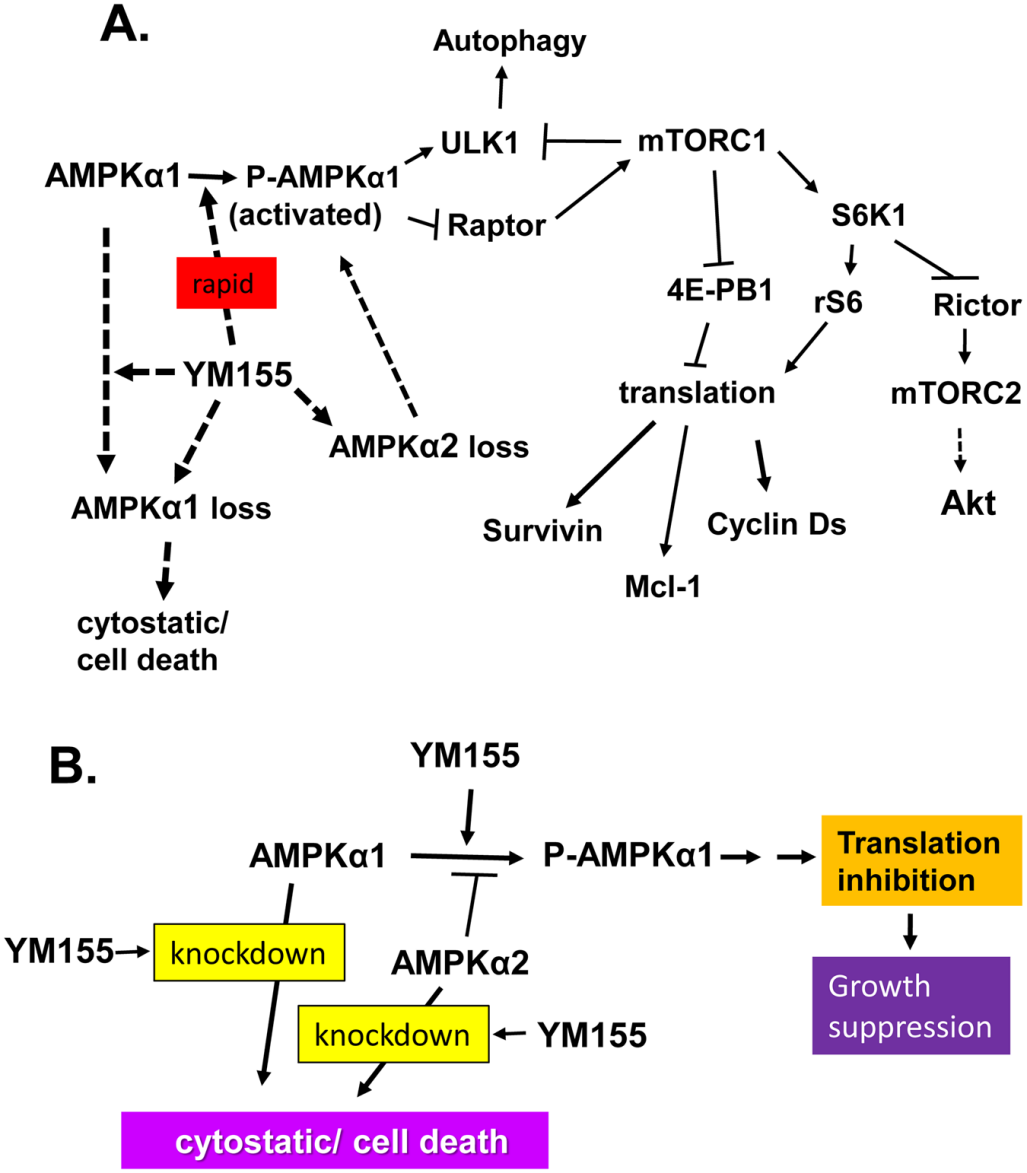
Summary of our model on the molecular action of YM155 in prostate cancer cells. Panel A represents the overall molecular steps in which our model supports that YM155 rapidly promotes the activation of AMPK and subsequent inactivation of mTORC1 and suppression of cap-dependent translation of proteins involved in cell cycle and survival. This model also illustrates that YM155 additionally promotes the loss of AMPKα1 or AMPKα2, and that such loss counter-intuitively promotes growth arrest and cell death. Panel B is a simplified version of panel A.

