# Supplementary material for: Early Cellular Responses of Prostate Carcinoma Cells to Sepantronium Bromide (YM155) Involve Suppression of mTORC1 by AMPK
Source: Sci Rep. 2019 Aug 8;9:11541. doi: 10.1038/s41598-019-47573-y (PMC6687778; doi:10.1038/s41598-019-47573-y)
Supplement: Supplementary file 1 — Supplementary Figures [file 41598_2019_47573_MOESM1_ESM.pdf]

Supplementary Information

Early Cellular Responses of Prostate Carcinoma Cells to Sepantronium Bromide (YM155) Involve Suppression of mTORC1 by AMPK

David Danielpour[1,2,3,*], Zhaofeng Gao[7#], Patrick M. Zmina[1#], Eswar Shankar[1#], Benjamin C. Shultes[1,4], Raul Jobava[7], Scott M. Welford[5,6], and Maria Hatzoglou[7]

Case Comprehensive Cancer Center Research Laboratories, The Division of General Medical Sciences-Oncology[1], Department of Pharmacology[2], Department of Biochemistry[4], Department of Radiation Oncology[5], and Department of Genetics and Genomic Sciences[7], Case Western Reserve University, Cleveland, OH 44106.

Department of Urology[3], University Hospitals of Cleveland, Cleveland, OH 44106

Department of Radiation Oncology[6], University of Miami, FL 33136

Running title: YM155 Suppresses mTORC1 via AMPK

#**Authors contributed equally**

Supplementary Figure S1

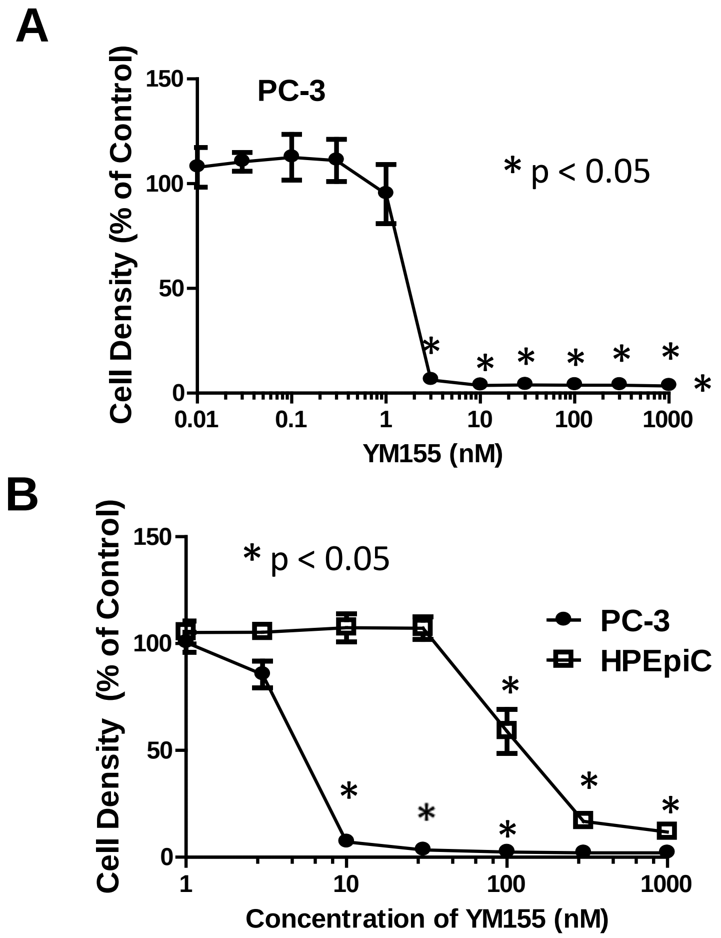

Supplementary Figure S1

Dose-dependent changes PC-3 and HEpiC cell density following 72 h treatment with various doses of YM155. A) PC-3 cells were plated in 24-well dishes at 5,000 cells/well/0.5 ml, the next day treated with YM155 and 72 h later cell growth was assessed with a crystal violet assay as described in the materials and methods. B) A side-by side comparison between the cytotoxic responses of YM155 on confluent cultures of PC-3 and HEpiC cells after 72 h drug addition, as assayed by in A.

Supplementary Figure S2

A.

Effect of YM155 on cell cycle in PC-3 cells after 24 hours

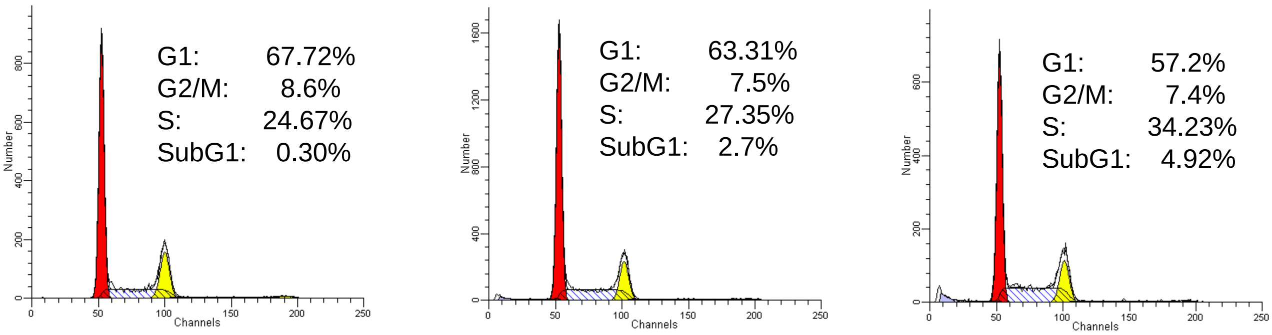

DMSO vehicle control

3 nM YM155

10 nM YM155

Effect of YM155 on cell cycle in PC-3 cells

B.

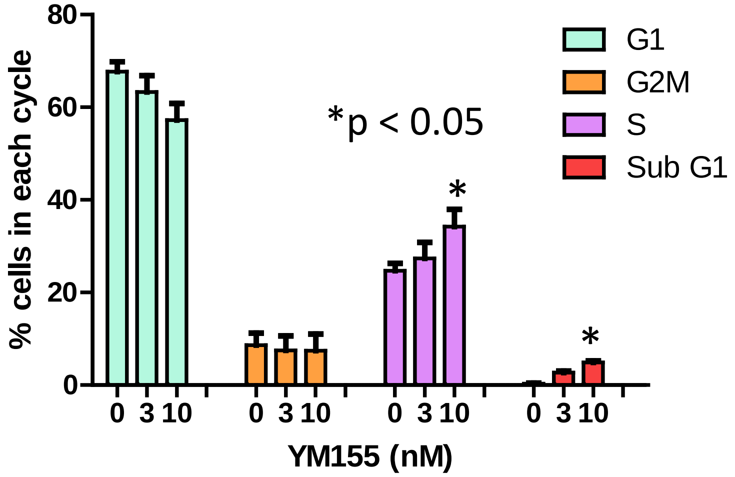

Cell cycle analysis was performed on PC-3 cells following treatment with vehicle or YM155 for 24 h. Cells were fixed with 90% methanol, stained with propidium iodide and analyzed by flow cytometry a described previously (Song et. al., EMBO, 25: 58-69, 2006). A) the y-axis represents the number of cells and the x-axis is the DNA content in per cell. B) Averages of the various phases of the cell cycle following YM155 treatment.

Supplementary Figure S3

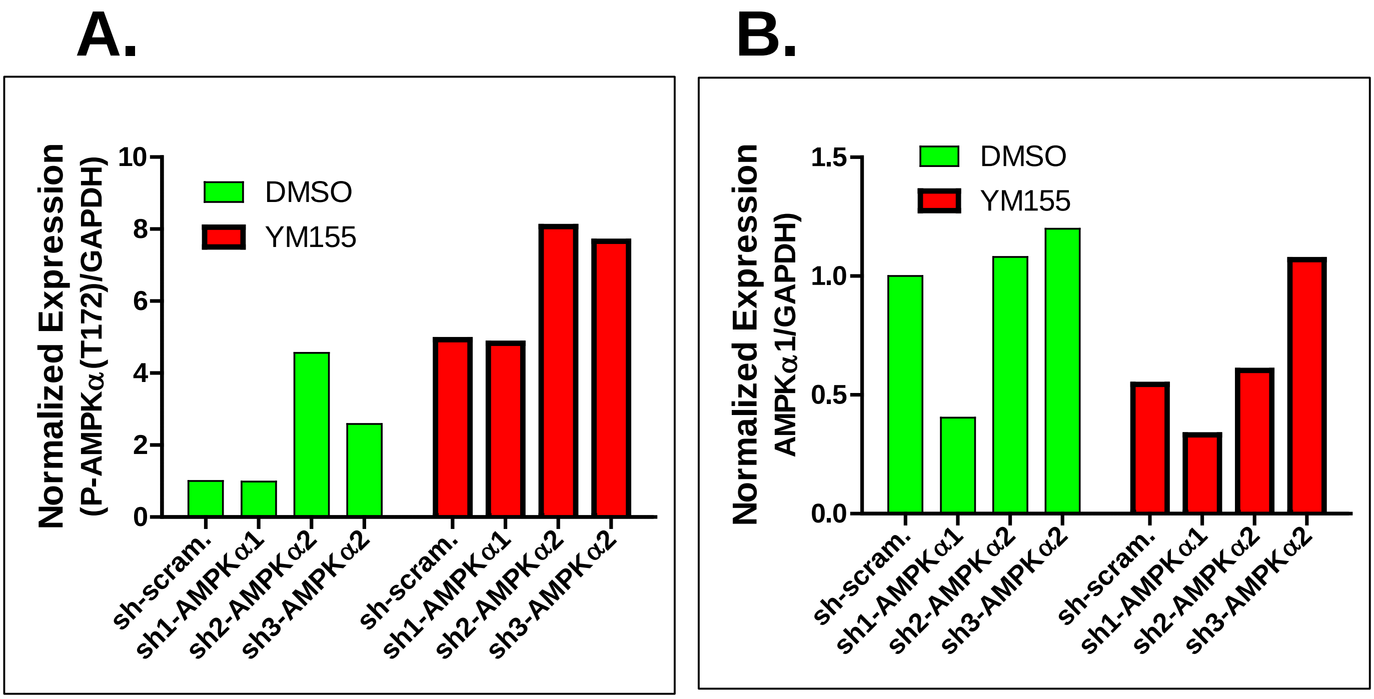

Supplementary Figure S2. Densitometry of Figure 9C by the Image J software for semi-quantitative analysis of P-AMPKα (T172) (A) and total AMPKα (B). Data are normalized to GAPDH loading control and expressed as normalized expression relative to untreated scrambled control.

Supplementary Figure S4

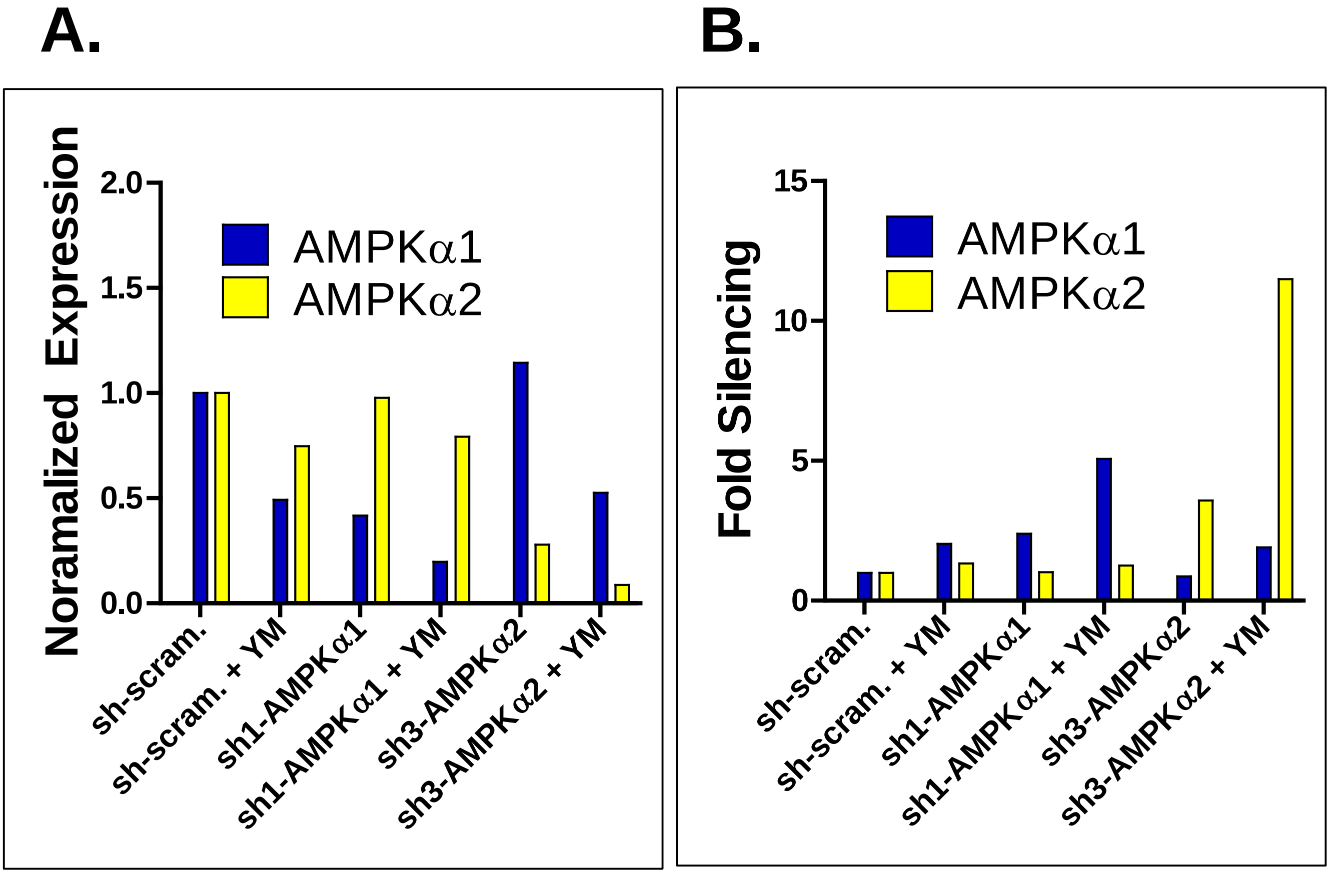

Supplementary Figure S3. Densitometry of Figure 9D by the Image J software for semi-quantitative analysis of AMPKα1 and AMPKα2. Data are normalized to β-actin loading control and expressed as normalized expression relative to untreated scrambled control (A), and fold induction over the scrambled untreated control (B).

Supplementary Figure S5

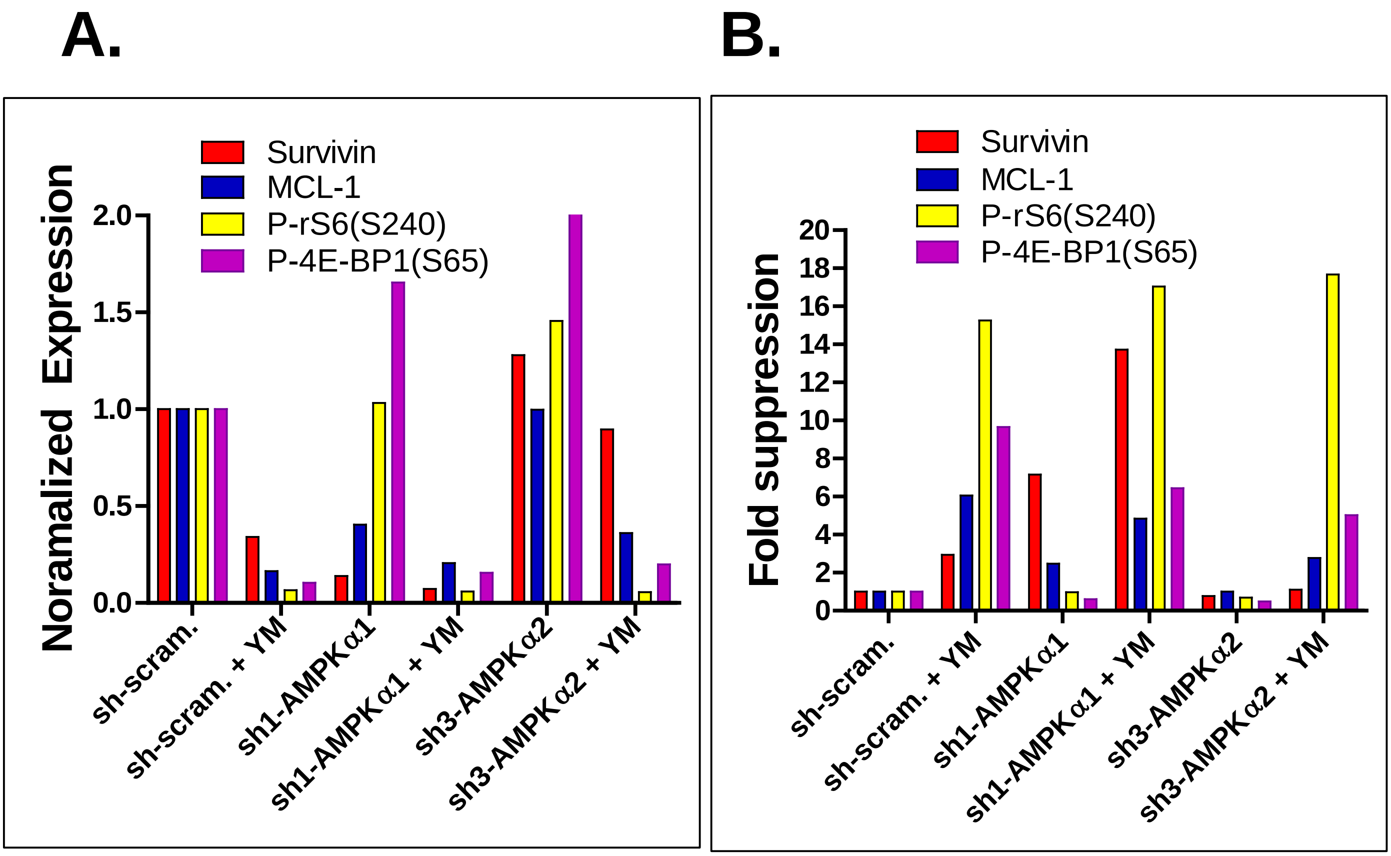

Supplementary Figure S4. Densitometry of Figures 9D and 9E by the Image J software for semi-quantitative analysis of Survivin, Mcl-1, P-rS6(240) and P-4E-BP1(S65). Data are normalized to their respective loading controls and expressed as normalized expression relative to untreated scrambled control (A), and fold induction over the scrambled untreated control (B).

Supplementary Figure S6

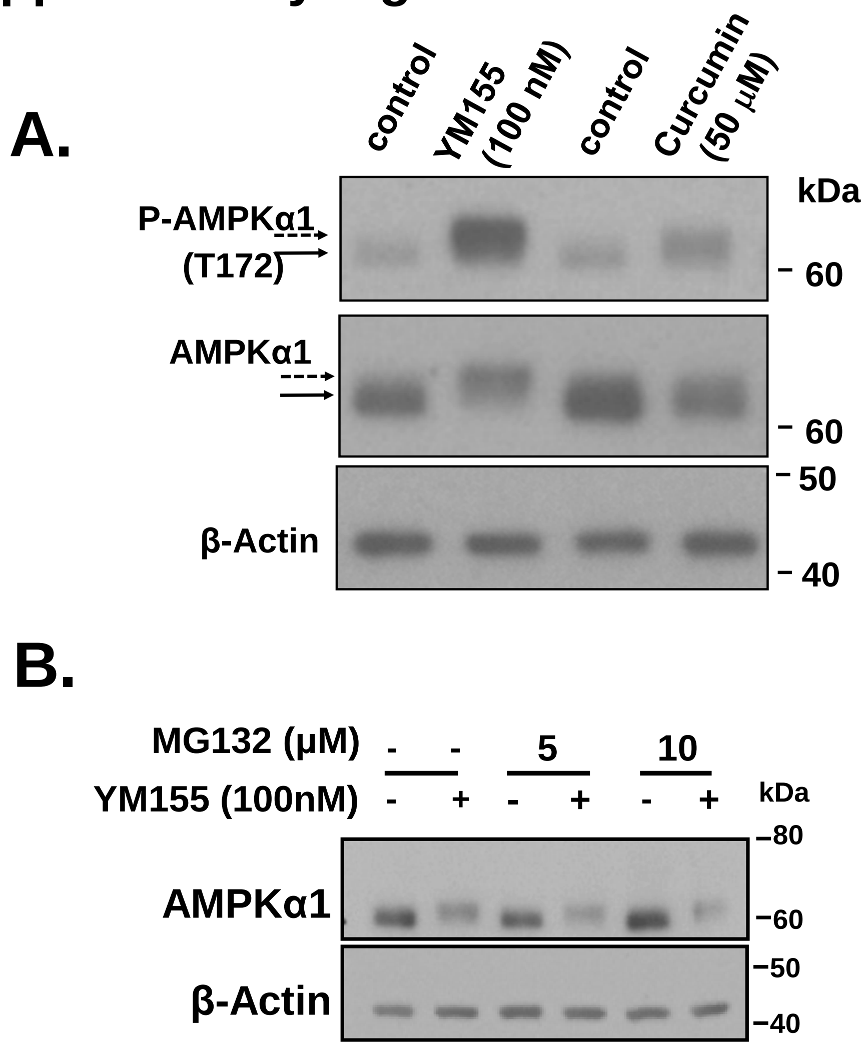

Supplementary Figure S5. Comparative effects of YM155 and Curcumin on total AMPKα1 and phospho-AMPKα levels in PC-3 cells after 4 hours of treatment, as assessed by Western analysis. **B)** Effect of MG132 on YM155 (4 h)-mediated loss of AMPKα1 in PC-3 cells was assessed by Western blot. Results shown are representative of 2 to 3 independent experiments.

Supplementary Figure S7

A.

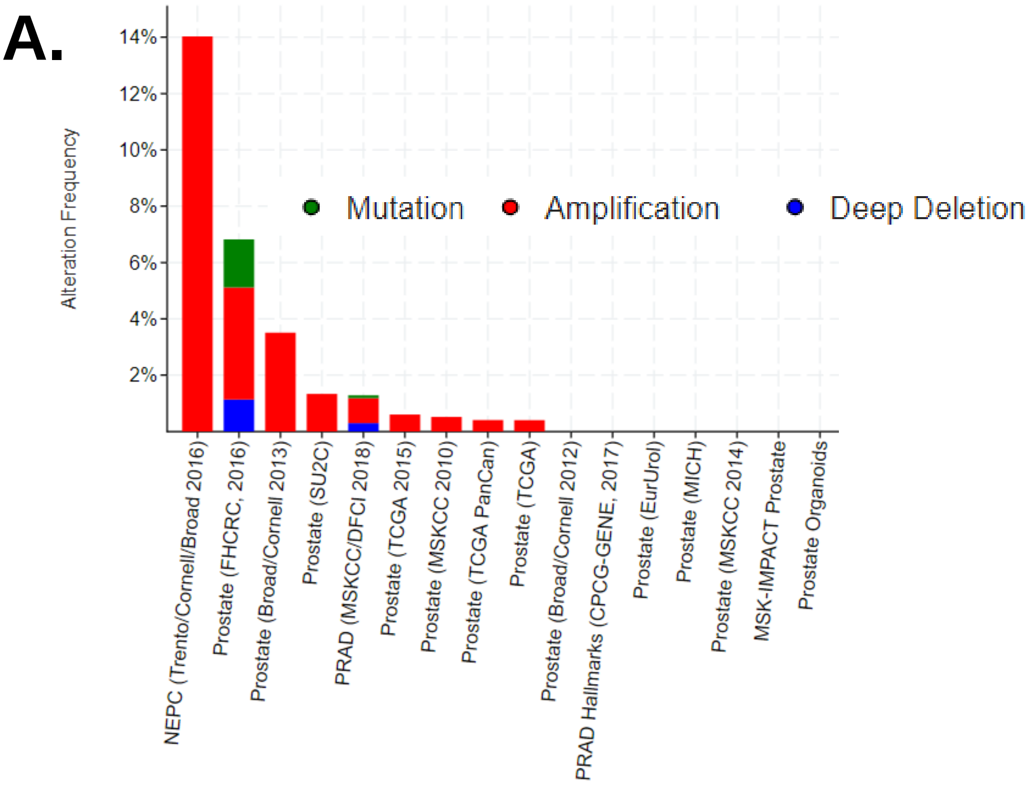

B.

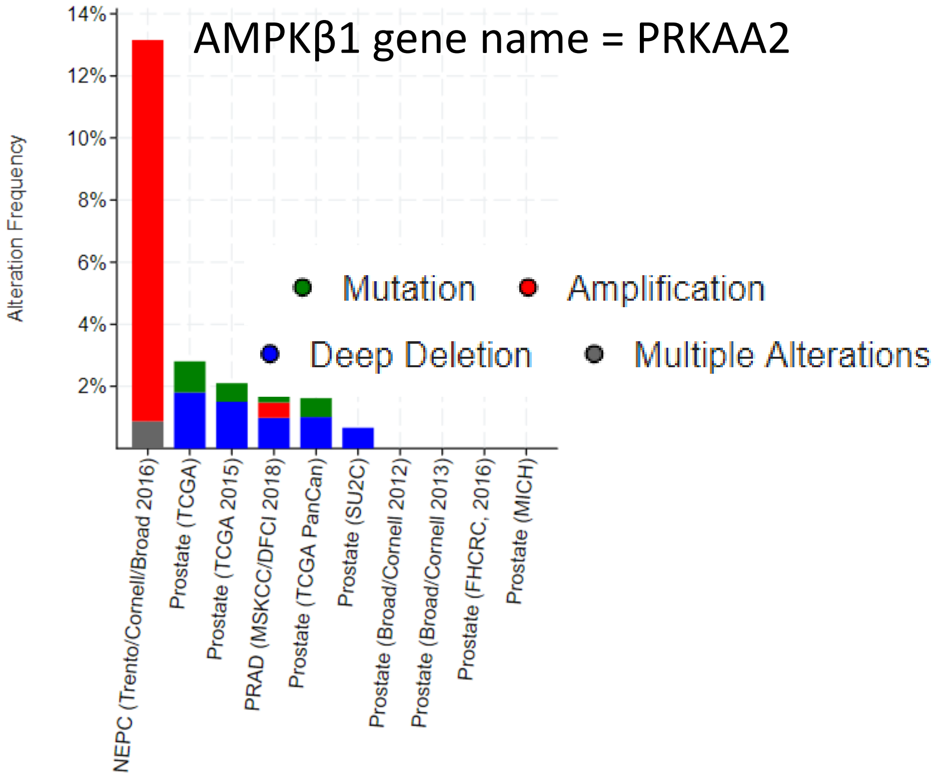

Supplementary Figure S6. Alterations in AMPKα1 **(A)** and AMPKα2 **(B) found** in the cBioportal (http://cbioportal.org) database support that AMPKα1 are AMPKα2 are significantly amplified in neuroendocrine prostate carcinomas.

Supplementary Figure S8

A.

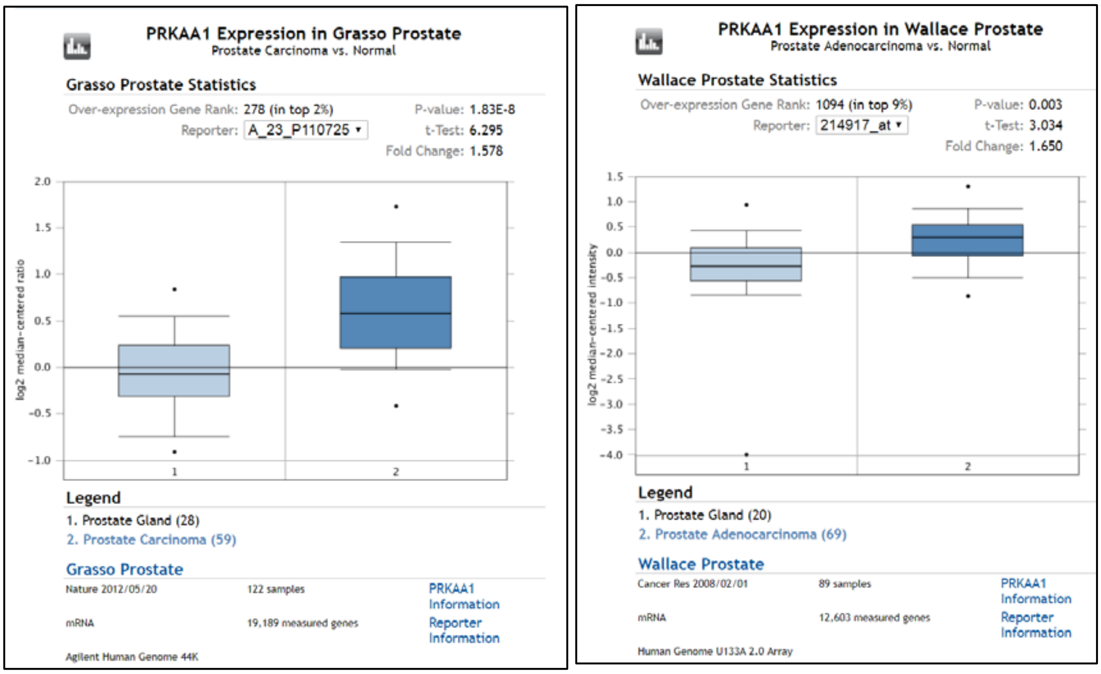

B.

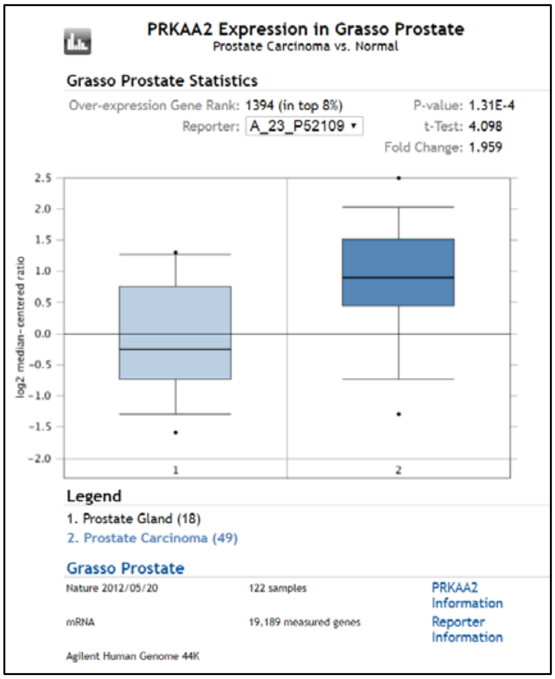

Supplementary Figure S7. Alterations in the expression of AMPKα1 **(A)** and AMPKα2 **(B)** **found in studies in Oncomine database** (https://www.oncomine.org/resource/login.html) support that AMPKα1 are AMPKα2 are significantly overexpressed in prostate carcinomas.
